# Effects of Trace Elements on Endocrine Function and Pathogenesis of Thyroid Diseases—A Literature Review

**DOI:** 10.3390/nu17030398

**Published:** 2025-01-22

**Authors:** Łukasz Bryliński, Katarzyna Kostelecka, Filip Woliński, Olga Komar, Agata Miłosz, Justyna Michalczyk, Jan Biłogras, Anna Machrowska, Robert Karpiński, Marcin Maciejewski, Ryszard Maciejewski, Gabriella Garruti, Jolanta Flieger, Jacek Baj

**Affiliations:** 1 Department of Forensic Medicine, Medical University of Lublin, Jaczewskiego 8b, 20-090 Lublin, Poland; lukbry2@gmail.com (Ł.B.); rush22235@gmail.com (F.W.); 2 Department of Correct, Clinical and Imaging Anatomy, Chair of Fundamental Sciences, Medical University of Lublin, Jaczewskiego 4, 20-090 Lublin, Poland; katarzyna.k@vp.pl (K.K.); olgakomar720@gmail.com (O.K.); agatamilosz95@wp.pl (A.M.); justynaelwiramichalczyk@gmail.com (J.M.); janbilogras@gmail.com (J.B.); 3 Department of Machine Design and Mechatronics, Faculty of Mechanical Engineering, Lublin University of Technology, Nadbystrzycka 36, 20-618 Lublin, Poland; a.machrowska@pollub.pl; 4 Institute of Medical Sciences, The John Paul II Catholic University of Lublin, Konstantynów 1H, 20-708 Lublin, Poland; m.maciejewski@pollub.pl (M.M.); ryszard.maciejewski@kul.pl (R.M.); 5 Department of Electronics and Information Technology, Faculty of Electrical Engineering and Computer Science, Lublin University of Technology, Nadbystrzycka 36, 20-618 Lublin, Poland; 6 Department of Precision and Regenerative Medicine and Ionian Area (DiMePre-J), University of Bari Medical School, 70124 Bari, Italy; gabriella.garruti@uniba.it; 7 Department of Analytical Chemistry, Medical University of Lublin, Chodźki 4A, 20-093 Lublin, Poland; jolanta.flieger@umlub.pl

**Keywords:** trace elements, micronutrients, macronutrients, metallomics, thyroid, hormones

## Abstract

The thyroid gland is an endocrine organ whose hormones enable the proper functioning of the organism. The normal function of this organ is influenced by internal and external factors. One of the external factors is trace elements. Trace elements in appropriate concentrations are necessary for the proper functioning of the thyroid. Fe, Cu, Mn, I, Zn, and Se are part of the enzymes involved in oxidative stress reduction, while Cd, Hg, and Pb can increase ROS production. Cu and Fe are necessary for the correct TPO synthesis. An imbalance in the concentration of trace elements such as Fe, Cu, Co, I, Mn, Zn, Ag, Cd, Hg, Pb, and Se in thyroid cells can lead to thyroid diseases such as Graves’ disease, Hashimoto’s thyroiditis, hypothyroidism, autoimmune thyroiditis, thyroid nodules, thyroid cancer, and postpartum thyroiditis. Lack of adequate Fe levels may lead to hypothyroidism and cancer development. The thyroid gland’s ability to absorb I is reversibly reduced by Co. Adequate levels of I are required for correct thyroid function; both deficiency and excess can predispose to the development of thyroid disorders. High concentrations of Mn may lead to hypothyroidism. Furthermore, Mn may cause cancer development and progression. Insufficient Zn supplementation causes hypothyroidism and thyroid nodule development. Cd affecting molecular mechanisms may also lead to thyroid disorders. Hg accumulating in the thyroid may interfere with hormone secretion and stimulate cancer cell proliferation. A higher risk of thyroid nodules, cancer, autoimmune thyroiditis, and hypothyroidism were linked to elevated Pb levels. Se deficiency disrupts thyroid cell function and may lead to several thyroid disorders. On the other hand, some of the trace elements may be useful in the treatment of thyroid diseases. Therefore, the effects of trace elements on the thyroid require further research.

## 1. Introduction

The thyroid gland is an organ that regulates key bodily functions through the production of hormones such as triiodothyronine (T3) and thyroxine (T4), which play a key role in early brain development; influence the growth and development of reproductive, nervous, and circulation systems; and affect somatic growth, bone maturation, and mRNA synthesis of over 100 proteins [1,2].

Many different factors can affect thyroid function, also leading to thyroid pathology. Due to the influence of the hormones thyrotropin-releasing hormone (TRH), and thyroid-stimulating hormone (TSH), the thyroid gland remains under the control of the hypothalamus–pituitary–thyroid axis (HPT). Infections, smoking, proper diet, and vitamin D levels are other potential factors. Their influence may disturb the function of the thyroid gland, leading to various thyroid pathologies. Among these pathologies, thyroid nodules and simple (diffuse) physiological goiter are the most prevalent thyroid conditions [3,4]. Being the most prevalent endocrine cancer, thyroid cancer accounts for less than 1% of all cancers registered in the United Kingdom [5]. In the United States, hypothyroidism and hyperthyroidism are the most common endocrine diseases [6].

Trace elements are also a factor in thyroid function and may therefore influence the occurrence of the pathologies mentioned above. Their source can be food as well as the external environment. While several micronutrients are essential for healthy thyroid cell physiology, many others have an effect on the thyroid gland and are regarded as endocrine-disrupting substances. Reactive oxygen species (ROS), which are connected to cytotoxicity, cell death, and malfunction, are produced by unbound iron (Fe) [7]. Cadmium (Cd) may also raise the risk of exposure of thyroid cells to oxidative stress [8]. The mercury (Hg) molecule, methylmercury (MeHg), the organometallic cation, has a similar impact on thyroid cells by preventing the formation of thioredoxin reductase, an enzyme that prevents oxidative cell damage [9]. Lead (Pb) accumulation can also negatively affect cells by causing oxidative stress, disruption of cellular energy production, and DNA damage [10]. In turn, other elements can have a beneficial effect on oxygen stress by reducing it. Superoxide dismutase (SOD) contains copper (Cu). As a component of the antioxidant defence, this enzyme shields thyroid cells from oxidative damage [11,12]. Manganese (Mn) acts as a cofactor in Mn-SOD and also influences the oxoreductive properties of thyroid cells. Zn has a similar function and is also necessary for the function of enzymes that prevent oxidative stress, such as Cu/Zinc (Zn) SOD [13,14]. The thyroid gland’s oxoreductive potential may also be affected by I (iodine). Glutathione peroxidase (GSH-Px), an enzyme involved in limiting the production of ROS, was shown to be expressed more in the thyroid gland when dietary I supply was increased in a rabbit model [15,16]. GSH-Px is a selenoprotein, so a normal concentration of selenium (Se) is required for it to function properly. Another selenoprotein, thioredoxin reductase, is also involved in protection against oxidative stress or inflammation [17]. One of the most important atoms in the thyroperoxidase (TPO) active site structure is Fe [18]. Intracellular Cu plays a key role in the regulation of proliferation and proper growth of follicular thyroid cells by regulating the expression of TPO and paired box gene 8 (*PAX-8*), a gene that has a regulatory function in thyroid cell development and differentiation [19,20]. Additionally, Zn contributes to the regulation of the production of several enzymes by being a component of the Zn finger proteins, which are engaged in promoter regulation and DNA binding [21].

Through the above-mentioned functions, trace elements can also influence the pathogenesis of thyroid diseases. In this review, we discuss the role of the individual trace elements Fe, Cu, Co, I, Mn, Zn, Ag, Cd, Hg, Pb, and Se in the pathogenesis of thyroid diseases such as Graves’ disease, Hashimoto’s thyroiditis, hypothyroidism, autoimmune thyroiditis, thyroid nodules, thyroid cancer, and postpartum thyroiditis. We describe the influence of their concentrations in the human body on the development of the above-mentioned diseases, the molecular mechanisms responsible for their development, and the potential role of elements in the treatment of some thyroid diseases. We also focus on their effects on thyroid hormone (TH) synthesis and metabolism.

## 2. Trace Elements and Hormone Synthesis and Metabolism

**Figure 1:**
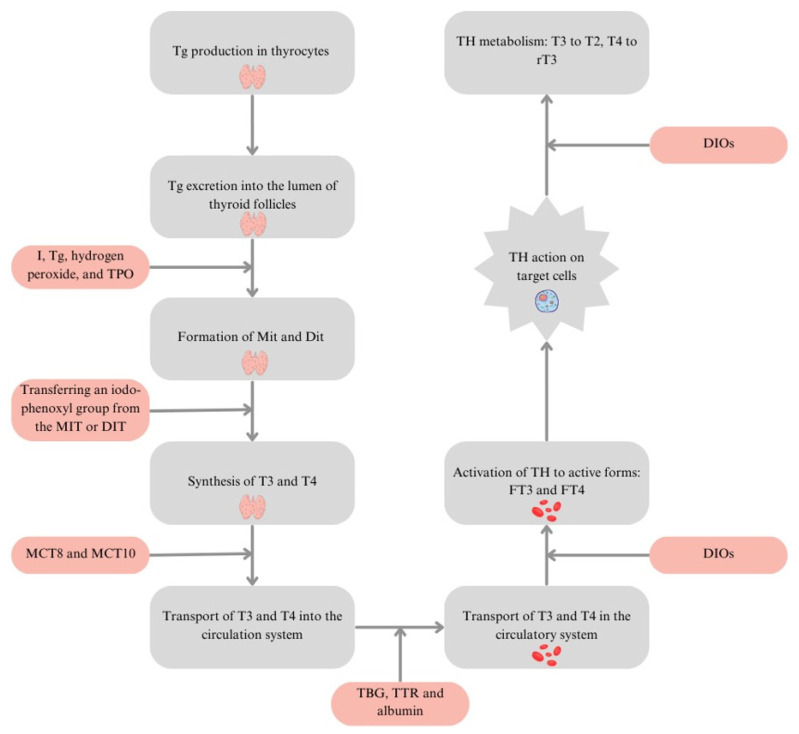
The synthesis and metabolism of thyroid hormones. DIO—deiodinase; Dit—diiodothyronine; FT3—free forms of triiodothyronine; FT4—free forms of thyroxine; I—iodine; MCT10—monocarboxylate transporter 10; MCT8—monocarboxylate transporter 8; Mit—monoiodothyronine; rT3—reverse triiodothyronine; T2—3,3′-diiodo-L-thyronine; T3—triiodothyronine; T4—thyroxine; TBG—thyroxine-binding globulin; Tg—thyroglobulin; TH—thyroid hormone; TPO—thyroperoxidase; TTR—transthyretin.

The synthesis of T4 and T3 occurs in the thyroid follicles. The TH synthesis requires I, which reaches the thyroid gland via the bloodstream. Thyrocytes produce thyroglobulin (Tg) and excrete it into the lumen of the thyroid follicles, followed by iodination of the tyrosine residues of Tg. This process depends on the iteration of I, Tg, H_2_O_2_, and TPO. Monoiodothyronine (Mit) and diiodothyronine (Dit) are formed. The synthesis of T3 and T4 is achieved by transferring an iodo-phenoxyl group from the MIT or DIT residue, called the “donor,” to the DIT residue, called the “acceptor” [22]. The formed T3 and T4 are transported into the blood circulation via the bidirectional transporters monocarboxylate transporter 8 (MCT8) and monocarboxylate transporter 10 (MCT10). In the blood, they are transported by the carrier proteins: thyroxine-binding globulin (TBG), transthyretin (TTR), and albumin. THs bound to these proteins are inactive and remain in dynamic balance with the active, free forms: free forms of triiodothyronine (FT3) and free forms of thyroxine (FT4). Deiodinases (DIOs) are enzymes engaged in the regulation of TH metabolism. DIO1 is involved in the conversion of T4 to T3 and rT3 and T3 to rT3 or T2, and DIO2 is involved in the conversion of T4 to T3 and T3 to T2. DIO3 is engaged in the conversion of T4 and T3 to rT3 and T2 (Figure 1) [23,24].

The HPT axis controls the thyroid’s production of hormones. In the paraventricular nucleus of the hypothalamus, hypophyseal neurons generate TRH. The anterior lobe of the pituitary gland contains thyrotrophs, which are stimulated to produce TSH by TRH. The thyroid follicular cells, which release T4 and T3, are the targets of TSH action [25].

One of the elements that is essential to the synthesis of TH is Fe. It enters into the composition of TPO, which is a heme-dependent enzyme. Deficiency of the element results in a decrease in its activity, with a consequent decrease in the synthesis of the thyroid hormone and compensatory increasing TSH levels and gland volume [26,27,28]. Fe deficiency affects TH levels [18]. Wang et al. [29] observed that pregnant women’s TH levels were correlated with Fe levels: serum ferritin (SF) and hemoglobin (Hb) levels were positively correlated with FT3 and FT4, but negatively with TSH [29]. The study conducted on a group of teenage Iranian girls with Fe deficiency revealed similar findings: Fe deficiency decreased plasma concentrations of T3 and T4 [30]. Fe deficiency anemia also results in a dysfunction of the HPT axis [7].

The other trace element, Cu, also influences the level of TH by stimulating T4 production and inhibiting excessive T4 absorption in blood cells by controlling calcium levels in the body [31]. The levels of Cu were positively associated with increased FT4 levels in males. No effect of Cu on TSH or FT3 was found [32]. A study by Jain et al. [33] showed that Cu was correlated with increases in FT4 and total T4 (TT4). Cu levels were positively correlated with total T3 (TT3) and TT4 in females [33]. On the other hand, a 2023 study of 1067 Chinese adults suggested that Cu levels were positively associated with FT3 and FT4 levels, respectively [34].

In research involving female plate painters who were exposed to Co, it was shown to affect the synthesis and metabolism of TH. It hinders the thyroid gland’s ability to absorb I. Co might also inhibit the extrathyroidal 5′-deiodination of T4 into T3, which may result in an increase in FT4 and a decrease in FT3 [35].

Since I is a component of their structure, it is necessary for the synthesis of THs [36]. Interestingly, excess I can inhibit TH synthesis. An investigation using a mouse model revealed that excess iodide reduced micro-RNA (mRNA) expression of genes involved in TH synthesis: thyrotropin receptor (TSHR), Tg, sodium/iodide symporter (NIS), TPO, *PAX8*, and NK2 homeobox 1 (*NKX2 1*). This effect of excess I can be achieved by regulating the expression of the X-box-binding protein 1 (*XBP1*) gene. *XBP1* represses the transcription of genes involved in the synthesis of THs, for which they are direct target genes [37]. This is a potential mechanism for the Wolff–Chaikoff effect (WCE), which involves decreased transport of I into the thyroid gland and decreased production of TH by thyroid cells when exposed to excess I [38].

Mn can affect DIO activity and therefore interfere with the T4 to T3 conversion. A study in a rat model showed that a diet extremely low in Mn led to an increase in hepatic 5′-deiodinase activity (5′DI) and an increase in relative T3 concentration [39].

Because Zn affects hepatic DIO activity, it may potentially have an impact on the conversion of T4 to T3: this enzyme’s activity decreases when Zn levels are low [40]. Furthermore, Zn affects the function of TTR, an enzyme that helps TH distribution through the body through extracellular fluids [41]. Zn, as a cofactor of the enzyme, is also involved in the processing of prepro-TRH to form TRH [42].

Silver (Ag) also affects the metabolism of THs. An investigation of laying hens showed that using silver nanoparticles (AgNP) increased the expression of iodothyronine DIO3 mRNA in the thyroid gland. Its overexpression leads to TH degradation. Meanwhile, in the liver, AgNP started to upregulate DIO2 [43].

Cd, as a result of inducing oxidative stress in thyroid cells, can affect their production of THs. Furthermore, Cd affects TH metabolism: through a mechanism of inhibition of hepatic 5′-monodeiodinase (5′-D) activity, it interferes with the T4 to T3 conversion. As a result, there is a decrease in T4 levels with no change in T3 concentrations. In addition to changes in 5′-D activity, the liver’s UGT activity is triggered by Cd exposure, which increases T3 and T4 metabolism and lowers their plasma concentrations. Due to Cd’s decomposition in the brain, it may also affect extrusion on the HPT axis [44]. Through several methods, Hg lowers the thyroid’s synthesis of T3 and T4. Hg in the form of MeHg interferes with TSH production. Inorganic compounds of Hg inhibit the production of TPO. In contrast, both MeHg and inorganic compounds inhibit Tg iodination [45]. According to a 2012 study, there is a relationship between Hg levels in the blood and levels of TT4, TT3, and FT3: they are inversely proportional [46]. A study of 137 male gold miners in Bibiani, Ghana, occupationally exposed to Hg showed that TSH was slightly elevated in the occupationally exposed group, but the change was not statistically significant. In contrast, T3 and T4 levels were significantly reduced in the exposed group. Blood Hg levels showed a positive correlation with working time [9]. On the other hand, Hu et al. in a meta-analysis investigating the effect of Hg on TH function showed that the presence of Hg in the blood could increase TSH and FT4 levels and negatively affect T4. The effect of Hg on blood TH levels requires further study [47].

TH synthesis is also affected by Pb exposure. By preventing deiodination and hence reducing T3 levels, Pb poisoning can obstruct the conversion of T4 to T3. Additionally, TSH is down while T4 is rising [48].

Se as an element is included in DIOs, which are selenoproteins: DIO1, DIO2, and DIO3 [24]. The impact of trace elements on the metabolism and synthesis of TH was summarized in Table 1.

## 3. The Role of Trace Elements in Thyroid Diseases

In thyroid disorders such as Grave’s disease, Hashimoto’s thyroiditis, hypothyroidism, autoimmune thyroiditis, thyroid nodules, thyroid cancer, and postpartum thyroiditis, trace elements including Fe, Cu, Co, I, Mn, Zn, Ag, Cd, Hg, Pb, and Se are crucial.

### 3.1. Iron

Fe is involved in immune processes (differentiation of lymphocytes and macrophages, the proliferation of B cells and T cells), erythropoiesis, reactions at the cellular level such as DNA replication and repair, mitochondrial function, and in enzymatic reactions as a cofactor. In tissues and body fluids, Fe forms complexes with proteins such as transferrin, and ferritin. Trace elements, including Fe, are one of the components of THs. Moreover, they are crucial for the metabolism and function of the thyroid gland itself and for the synthesis of its hormones [31,49]. Fe deficiency manifests mainly as anemia and is regulated by cytokines and acute-phase proteins. Elemental deficiency is found in infections, tumors, and autoimmune diseases. Both Fe deficiency and excessive Fe accumulation can lead to cytotoxicity, so physiologically controlled Fe metabolism plays a key role in its critical role in the catalytic processes of proteins and matrix metalloproteins (MMP) enzymes [50,51,52]. Fe is involved in cell division, which can be exploited by pathogens and has a role in the subsequent development of infection [53,54]. It is involved in the stimulation of TPO activity [52,55]. Adequate Fe levels are necessary for the correct function of TPO, which function was described in chapter 2 [27,28]. A study of the link between Fe levels and TH indicates that individuals with thyroid dysfunction had significantly increased Fe concentrations compared to the healthy control group, which suggests that Fe is engaged in the pathogenesis of thyroid disturbance [56]. On the other hand, other data do not support any relationship between levels of both Fe and Cu and diseases of the thyroid [57].

#### 3.1.1. Iron and Graves’ Disease

A study of patients with Graves’ disease with associated ophthalmopathy, made by using magnetic resonance imaging (MRI) and clinical scale assessment, showed increased Fe deposition in the brain, which could account for visual, emotional, and cognitive deficits [58]. Graves’ disease may be associated with Fe deficiency anemia. Patients with Graves’ disease and Fe deficiency anaemia are at additional risk of autoimmune atrophic gastritis [59].

#### 3.1.2. Iron and Hashimoto’s Thyroiditis

Patients with Hashimoto’s thyroiditis and subclinical hypothyroidism have been reported to have lower serum Fe levels as well as the incidence of Fe deficiency compared to controls. Studies have shown that anemia may have the effect of reducing plasma T3 and T4 levels while increasing TSH levels. Fe deficiency may decrease TPO activity—the most frequent cause of thyroid dysfunction [2,60,61]. Hashimoto’s disease is associated with anemia and deficiency of vitamin D. It has been shown that 58% of patients with Hashimoto’s thyroiditis suffer from Fe deficiency anemia and 75% from vitamin D deficiency [62,63]. It has been shown Hashimoto’s disease patients often have lower serum Fe levels in Fe deficiency anaemia. They are often accompanied by other autoimmune diseases such as gastritis and celiac disease, among others, causing problems in the absorption of the element [61].

#### 3.1.3. Iron and Autoimmune Thyroiditis

Autoimmune thyroid diseases (AITDs) have a multifactorial pathogenesis and are associated with multifactorial interactions. It is thought that Fe deficiency may reduce the activity of TPO and 5′-deiodinase, as well as inhibiting T3 with its nuclear receptor, thereby affecting the slower release of T3 from serum. Deficiency increases the risk of autoimmune diseases [64]. It has been found that Fe levels were reduced in people with autoimmune thyroiditis [65]. It has been shown that reduced blood Fe levels alongside magnesium are associated with an increase in antibodies to thyroiditis and may contribute to autoimmune thyroiditis in women of childbearing age. Elevated serum Fe levels were associated with lower autoantibodies against thyroid peroxidase (TPOAb) and autoantibodies against thyroglobulin (TgAb) concentrations, with a greater effect on TPOAb score than on TgAb. Age, gender, body mass index (BMI), smoking, alcohol intake, iodized salt, HbA1c, total cholesterol (TC), and triglycerides (TG) were taken into account. Comparing the prevalence of autoimmune diseases in postmenopausal women and men, no differences were found. The opposite results were obtained when comparing the aforementioned groups to women of reproductive age, in whom the prevalence of autoimmune diseases was higher. The study suggests that adequate Fe supplementation may help prevent autoimmune thyroiditis in women of reproductive age [66]. Several studies have shown that AITD may be linked with intestinal microflora, which is associated with modifications in intestinal permeability barrier function. This affects the absorption of, among other things, Fe [67]. Patients with autoimmune thyroiditis had significantly lower serum Fe and Mn concentrations. Logistic regression and restricted cubic regression analysis showed that there was a non-linear association between Fe and TPOAb and TgAb positivity. It has been suggested that low magnesium and Fe levels are risk factors for the development of autoimmune thyroiditis in women of childbearing age [66].

#### 3.1.4. Iron and Thyroid Nodules

Although it has been suggested that decreased levels of trace elements are related to the risk of nodular goiter, a study of 25 patients found no change in Fe levels between those with nodular goiter compared with the control group [68]. On the other hand, a study that included more than 1000 participants with thyroid nodules in areas with normal I levels found reduced Fe levels in patients [69].

#### 3.1.5. Iron and Thyroid Cancer

There is a need for further research to estimate the effect of trace elements on the aggressiveness and progression of papillary thyroid cancer (PTC) [70]. Most studies confirm the association between Fe and the occurrence of thyroid cancer by facilitating tumor cell proliferation. Fe levels may facilitate tumor cell proliferation through a relationship with hepcidin secreted by thyroid cancer cells, thereby increasing intracellular Fe concentrations [71,72]. By assessing the effect of trace elements on the aggressiveness characteristics of PTC, it was shown that elevated serum Fe levels were related to a lower risk of capsular infiltration, tumor growth > 1 cm, and the occurrence of advanced T-stage (T3/4a/4b) [70]. Treatments are being sought for PTC using the induction of ferroptosis with artesunate (ART), where its resistance has been identified. Ferroptosis is a form of Fe-dependent programmed cell death [73]. By demonstrating the carcinogenic activity of the ubiquitin-specific peptidase 10 (UPS10) in vivo in nude mice, it was noted in tetrahydrocannabinolic acid (THCA) thyroid cancer that UPS10 suppressed ferroptosis of THCA cells. Drugs targeting UPS10 expression are being sought [74]. The Fe-treated follicular thyroid cancer (FTC) cell line 133 was shown to induce increased ferroptosis. The anaplastic carcinoma cell line 8505C was found to have increased ferroptosis resistance [75]. In a study of genes associated with Fe metabolism, three were found to be associated with predictive and clinicopathological features of PTC [76]. Glutathione peroxidase 4 (GPX4), identified as a molecule that regulates ferroptosis in thyroid cancer tissues, is markedly elevated. Its overexpression results in inhibition of ferroptosis and further oncogenesis [77].

### 3.2. Copper

Cu is an element found in plasma in the form bound to ceruloplasmin or albumin and is involved in immune processes [31,50,78]. Deficiency can cause inflammatory reactions in the body. Adequate levels of Cu are required for the body to function properly: occurrence in excess is harmful, but small amounts are necessary for many physiological processes including respiration, maintenance of oxidative balance, and synthesis of TH [27,49,50,78]. Cu is mainly found in the liver, bones, and muscle. In animal model studies, it has been found that Cu deficiency leads to thymic shrinkage, spleen enlargement, neutropenia, and reduced T-lymphocyte count [50,51]. Elevated levels of Cu are found in the body in diabetes, infections, and cancer with no clear evidence of an effect on disease progression or treatment [31,50,79].

#### 3.2.1. Copper and Graves’ Disease

A correlation of levels of serum Cu with various diseases including Graves’ disease is being sought. Increased serum Cu levels appear to be positively associated with the occurrence of hyperthyroidism, taking into account factors such as gender, age, and BMI, as well as higher levels of specific antithyroid antibodies [80]. In subjects with stable euthyroidism or subclinical post-treatment hyperthyroidism with pre-existing Graves’ disease, there was no difference in Cu levels. It has been reported that low Cu levels may contribute to orbitopathy in Graves’ disease [80,81].

#### 3.2.2. Copper and Hashimoto’s Thyroiditis

By regulating calcium levels in the body, Cu plays a supporting role in the synthesis and absorption of TH. TSH stimulation, which depends on phospholipid synthesis, also indirectly depends on Cu. The general antioxidant imbalance is associated with thyroid disorders such as thyroid cancer, Hashimoto’s disease, dysthyroidism, and goiter [31]. Patients with Hashimoto’s disease showed higher serum Cu levels. At the same time, the ratio of Cu to Zn was lower, and the ratio of Cu to Se was equal [82]. In contrast, patients with Hashimoto’s thyroiditis 45–50 years ago did not have elevated Cu or Zn levels [83]. TH is involved in oxidative metabolism. The SOD present in the thyroid gland has been shown to catalyse the conversion of H_2_O_2_ to H_2_O and oxygen. The enzyme itself has a Cu/Zn complex in its active center, so proper levels of the above-mentioned elements may affect antioxidant protection. However, this study did not show an association between Cu and Zn levels and markers of oxidative stress and serum levels of Hashimoto’s thyroiditis-specific antibodies TPOAb and TgAb [81,83].

#### 3.2.3. Copper and Hypothyroidism

Comparison of trace element concentrations in hypothyroid patients yields conflicting results [65]. It has been shown that low Cu levels can be accompanied by hyperthyroidism and high levels by hypothyroidism [2]. High Cu levels have been associated with subclinical hypothyroidism in pregnant women [84]. A study conducted on a group of 43 patients with subclinical hypothyroidism in I-rich areas compared Cu levels before and after thyroid replacement therapy. No association was found between thyroid function and levels of the element after the therapy [85]. An analysis of the health status of 93 individuals showed that serum Cu levels were increased in those struggling with hypothyroidism [86]. Numerous studies found no significant statistical differences between serum levels of Cu in hyperthyroid subjects and those recorded in healthy individuals [65]. In a study of 160 individuals, including 80 with hypothyroidism, it was found that Cu concentrations were not statistically significant, although they were higher in healthy individuals. In addition, it was noted that reduced Zn levels promote Cu absorption from the gut. It was noted that the correlation between Cu, FT3, and FT4 was positive, whereas the correlation between Cu and TSH was negative [87]. A study of 68 hypothyroid patients showed that those with hypothyroidism had higher Cu concentrations. No correlation was seen between Cu levels and hormone levels. Among other things, serum Cu concentrations were influenced by the patient’s gender, smoking, and diet [56]. In contrast, another study did not confirm that hypothyroidism is associated with low serum Cu concentrations [88]. A study of 84 patients showed that in children with congenital hypothyroidism, there is a close relationship between serum Cu levels and THs in the postnatal period. Inadequate nutrition in children with this condition may cause Cu deficiency. Cu and thyroid hormone levels are closely related, and there is a positive correlation between them during early childhood [89].

#### 3.2.4. Copper and Autoimmune Thyroiditis

A comparison of hypothyroid and autoimmune thyroiditis patients conducted on 323 patients showed that serum Cu levels were comparable in both groups [90].

#### 3.2.5. Copper and Thyroid Nodules

The results involving the association between the thyroid nodules’ presence and trace element levels are inconsistent. It has been suggested that an increased risk of developing nodular goiter is associated with low levels of elements including Cu [91]. In contrast, another study found higher Cu concentrations in patients with multinodular goiter [68]. In a study conducted in areas with adequate I in the environment, involving 1048 participants with thyroid nodules, an imbalance of Cu, Zn, and Mn was shown to be associated with the presence of nodules. Participants with thyroid nodules had increased levels of the trace metals Cu, Mn, and Zn, but decreased levels of Fe [69]. Only women showed a positive correlation between serum Cu concentration and the incidence of thyroid nodules. Likely, differences in hormone management, genotype, and different metabolisms of the two sexes cause differing sensitivity and response to trace element levels in this Cu [92]. A study of 6480 Chinese euthyroid patients aimed to demonstrate the associations of metallic elements with the occurrence of thyroid nodules and their nature. Cu levels were shown to be positively associated with thyroid nodules in women with a mean age of 40.6 years. Women and the elderly were more likely to have malignant and non-malignant nodules [93]. In the nodular goiter group, serum Cu levels were comparable to the control group, and Zn and Se levels were higher than in healthy subjects. There was no correlation of trace elements with levels of TH levels or thyroid volume. Regardless of the number of thyroid nodules, Cu, Zn, and Se levels did not vary [94]. Plasma Cu levels in subjects, with normal dietary I satisfaction, were shown to be positively correlated with FT4. Trace element levels did not affect thyroiditis. Cu levels were associated with T4 or free TSH in both sexes. Of the trace elements, only plasma Cu levels were marginally correlated with the occurrence of thyroid nodules [11]. A placebo-controlled, double-blind study tested the safety and efficacy of non-invasive therapy for benign thyroid nodules. During each follow-up, in addition to recording nodule dimensions, plasma levels, T4, and Cu were assessed. In both hormone levels and serum Cu levels, no significant differences in values between measurements were observed [95]. Studies in animal models and humans suggest that trace elements including Cu seem to be usable as a marker in the differential diagnosis of thyrotropin-secreting adenoma (TSHoma) and resistance to thyroid hormone β (RTHβ) [96].

#### 3.2.6. Copper and Thyroid Cancer

There are a growing number of reports that indicate that Cu may contribute to thyroid cancer tumour development through cell proliferation, angiogenesis, and carcinogenesis; also, abnormal serum Cu levels may be associated with cancer recurrence. On the other hand, its possible use in targeted anticancer therapy is being investigated. A v-raf murine sarcoma viral oncogene homolog B1 mutation involved in Cu-dependent oncogenic signaling has been found to be the most frequent thyroid cancer genetic alteration, indicating the need to identify details of how cuproptosis affects the development of thyroid cancer cells [97,98]. Cuproptosis is a form of cell death dependent on intracellular Cu accumulation and upstream regulation of ferredoxin-1 (*FDX1*). It was shown that *FDX1* expression was lower in samples with thyroid cancer-containing material compared to healthy tissue. The study used esclomol (ES), an inducer of cuproptosis. ES inhibited the growth of thyroid cancer cells by increasing cuproptosis while simultaneously increasing Cu levels and *FDX1* expression compared to control groups [99,100]. *BRAF V600E* as the most common alteration in thyroid cancer is used in the targeted treatment of thyroid cancer. It is indicated that Cu enhances the antitumor efficacy of the applied disulfiram (DSF). This study assessed the effect of DSF/Cu on thyroid cancer cells and the response of these cells to the BRAF kinase inhibitor PLX4032. It was shown that DSF/Cu had a more effective effect in inhibiting thyroid cancer cell proliferation with the *BRAF V600E* mutation compared to the use of disulfiram alone, killed thyroid cancer cells through ROS-dependent suppression of the mitogen-activated protein kinase/extracellular signal-regulated kinase (*MAPK/ERK*) and phosphatidylinositol 3-kinase/protein kinase B (*PI3K/AKT*) pathways, increased the response of cancer cells with the *V600E* mutation to PLX4032 through ROS-dependent inhibition of human epidermal growth factor receptor-3 (*HER3*) and *AKT*, and decreased feedback activation of the *MAPK/ERK* and *PI3K/AKT* pathways [101]. In the treatment of PTC, Cu was used. For kinase activity to be inhibited with a cost-effective Cu-based chelator tetrathiomolybdate (TM), the antitumor Pefficacy of tetrathiomolybdate in PTC with the *BRAF V600E* mutation was investigated. TM inhibited mitogen-activated protein kinase (*MEK1/2*) activity and transformed PTC cell growth [102]. The study was designed to identify the effects of metal oxide nanoparticles (MONPs) on human thyroid cancer cells (ML-1), as well as rat medullary thyroid cancer (MTC) cells (CA77). After treatment with the use of ZnO and CuO, reduced cell viability was observed in both lines. However, there were no significant changes in ROS levels in the aforementioned cell lines after ZnO and CuO treatment while an increase in cell apoptosis was noted, suggesting ROS-independent but apoptosis-dependent death of thyroid cancer cells linked with changes in a number of signaling cascades [103].

The study from 2011 showed that PTC patients had higher serum Cu levels compared to controls and Hashimoto’s disease patients. Cu as a cofactor of cellular and extracellular isoforms of SOD has a role in defense mechanisms against oxidative damage [12]. It was shown that in the group of thyroid cancer patients, Cu/Zn ratio was higher [31]. High Cu intake causes increased thyroid volume, hyperthyroidism, hypothyroidism, autoimmune diseases, and cancer. Low Cu intake does not cause an increase in thyroid volume, causes hyperthyroidism or hypothyroidism, but does not cause autoimmunity and cancer [2]. Other data indicate that elevated levels of trace metal mixtures including Cu may be associated with thyroid cancer mortality in women [104]. Patients with thyroid cancer had elevated serum Cu levels compared to controls. Furthermore, Cu is elevated in the group of malignant thyroid cancer patients and reduced in the group with benign thyroid cancer [69]. It is thought that Cu may initiate angiogenesis in tumor cells; however, one study conducted in vivo did not find an association between Cu levels and thyroid cancer [72]. Cells with high aldehyde dehydrogenase (ALDH) activity have been shown to represent a population of cancer stem cells (CSCs) in differentiated thyroid carcinomas (DTCs), which can be used to apply DSF (ALDH inhibitor). DFS/Cu has been shown to act on cancer stem cells (CSCs) in tumors; for this reason, an analysis of the effect of the therapy on FTC and PTC cells was performed in which it was shown that the DSF/Cu complex inhibits thyrosphere formation while inhibiting CSC cell activity, potentially indicating the future use of this compound in the treatment of thyroid cancer [105]. A study comparing the levels of trace elements (i.e., Cu, Zn, Fe, and Mn) in MTC cells to a control group was performed with simultaneous analysis of MMPs and tissue inhibitors of matrix metalloproteinases (TIMPs). It was shown that in cells presenting MTC, Zn, Fe, and Mn levels decreased while Cu levels increased compared to control samples [106]. The effect of a therapy for anaplastic thyroid carcinoma (ATC) using synthesised 131I-labeled CuS nanoparticles modified with BSA (131I-BSA@CuS) was investigated. The study was performed on subcutaneous tumors of ARO cells, which are an ATC cell line. In vivo tests demonstrated the efficacy of the synthesized nanoparticles in anti-tumor therapy, providing hope for the application of the therapeutic line in a clinical setting [107]. Markers are being sought for the thyroid cancer diagnosis. Changes in Cu concentration in thyroid cancer have been observed. There are reports that the ratio 65 Cu/63 Cu (δ 65 Cu) may in the future act as a cancer biomarker. According to the study, plasma Cu concentrations in PTC patients were higher compared to the healthy control group. The plasma δ 65 Cu levels of the patients were lower compared to the healthy group, while it was noted that thyroid tumor tissues were characterized by high δ 65 Cu values. The study may suggest the association and usefulness of Cu as a marker for thyroid cancer [108]. Studies in animal models using radioactive Cu sulphide nanoparticles have demonstrated anti-tumor effects based on tumor growth and animal survival. In human experiments, delayed tumor growth was observed by increasing the therapeutic efficacy of radiotherapy with CuS-based photothermal therapy [109].

### 3.3. Cobalt

Co is a micronutrient that is a component of vitamin B12. Its sources can include eggs, dairy products, and green vegetables [110]. Co can be used in the metallurgical industry in the production of dyes, tyre glue, batteries, and accumulators [111].

#### 3.3.1. Cobalt and Graves’ Disease

Co is the element used to treat severe Graves’ ophthalmopathy (GO) in the form of Co irradiation. It is relatively safe and effective, with an overall response rate of 96% and a patient satisfaction rate of 98% [112]. Concomitant use of glucocorticosteroids prevents the development of acute side effects. There was no statistically relevant increase in the prevalence of cataracts, and cataract formation may be related to the aging process rather than the therapy itself [112,113]. The risk of developing radiation retinopathy is relatively low, and patients with diabetes and hypertension, which can be a relative contraindication to the procedure, are at greater risk of developing it [113]. There are difficulties in assessing the risk of developing secondary cancer, due to the long latency period of radiation-induced tumors, but in 20-year follow-ups of patients undergoing Co irradiation, no increased incidence of head and neck cancer was found [112,113,114]. There is a need for future research investigating the possible impact on disease progression and the use of Co in other forms of treatment.

#### 3.3.2. Cobalt and Hypothyroidism

Co can cause the development of hypothyroidism, because it causes a reversible decrease in I uptake in the thyroid gland [115]. The progression of hypothyroidism in head and neck cancer patients being treated with an external beam using 60 Co or a linear accelerator has been described. The incidence of hypothyroidism in this group of patients was 8% at 3 months after radiation therapy and increased to 14% at 6 months and was highest among patients treated with the two-dimensional technique [116]. The development of hypothyroidism can also result from the release of Co into the bloodstream from mounted polyethylene-metal total hip arthroplasty (THA) prostheses of the femoral head with Co chromium [115,117,118,119]. Two cases of asymptomatic patients with hip replacements and elevated blood Co and chromium levels were described. Their levels were decreased as a consequence of the use of *N*-acetylcysteine [120]. Other chelating agents such as ethylenediaminetetraacetic acid (EDTA), 2,3-dimercaptopropane-1-sulfonate (DMPS, unithiol), and dimercaprol may also be used [121,122,123]. Hypothyroidism, which can be caused by Co toxicity, increases the risk of contact allergy to Co. A study investigating the contact allergy risk in dental patients who were treated with nickel- and cobalt-containing materials found that the risk of contact allergy increased three-fold. In the case of nickel and Co, there is a phenomenon of conjugated allergy in which about one third of patients allergic to nickel are allergic to Co, and isolated reactions to Co without nickel and chrome are rare. Further studies on a larger group are needed to determine exactly which allergens are associated with the occurrence of hypothyroidism and whether there is a true cross-allergy between nickel and Co [124].

#### 3.3.3. Cobalt and Autoimmune Thyroiditis

There is a dearth of studies identifying the association between Co and the development of autoimmune thyroiditis in the literature. There is one case study of a woman who developed systemic contact dermatitis (SCD) (type IV hypersensitivity) and hypothyroidism in the course of autoimmune inflammation of the thyroid gland (type III reaction) with non-steroidal anti-inflammatory drugs (NSAIDs) hypersensitivity (type I reaction) as a result of wearing Co-containing jewellery. This is a very rare phenomenon of co-occurrence of several types of immune responses and may be related to monomeric immunoglobulin E (IgE), which may present a heterogeneous function as an autoreactive antibody against self-antigens as well [125]. Current data describing the effects of Co on thyroid nodules development are lacking. A 1995 study describing concentrations of various elements in healthy thyroid tissue and benign and malignant thyroid nodules indicates elevated levels of Co in benign and malignant thyroid nodules [126].

#### 3.3.4. Cobalt and Thyroid Cancer

A case-control study carried out in China showed a negative influence of Co on the development of thyroid cancer [127]. Another study shows that Co reduces lymph node metastasis and the risk of capsular invasion in PTC [70]. In the case study reported here, a patient with PTC achieved a complete response to external beam radiotherapy (EBRT) with Co 60 teletherapy applied to the entire neck at a dose of up to 44 Gy in 22 fractions for 4.5 weeks. After completion, another booster dose of 16 Gy in a smaller volume was administered. The authors of this study also point to the need for further research to evaluate EBRT as a curative therapy compared to other modalities [128]. For FTC, Co can also be used for external beam radiation treatment. When 60 Co was externally irradiated to cells of the WRO cell line of differentiated thyroid carcinoma (DTC) at doses below 8.3 Gy, there was no significant difference from controls, while higher doses of 60 Co resulted in a decrease in cell number starting on the sixth day after irradiation for the higher doses of 20 and 40 Gy and on the eighth and tenth days for doses of 10 and 4 Gy. While 60 Co has high penetration power and may be an adjunct to irradiation therapy with I-131, further studies are needed [129].

### 3.4. Iodine

I is a major component of TH and is a regulator of thyroid function. Iodized salt, fish, seaweed, seafood, and dairy products are dietary sources of I [130]. I consumption should be 250 µg per day for pregnant women, 90 µg per day for babies and children under six, 120 µg per day for children aged six to twelve, and 150 µg per day for adolescents and adults from the age of thirteen until adulthood [131].

#### 3.4.1. Iodine and Graves’ Disease

Many nations have used universal salt iodization (USI) to lessen the adverse effects of insufficient I supplies. However, excessive I intake also negatively affects thyroid function [132]. Excessive I results in the development of the iodine-based phenomenon (JBP), also known as iodine-induced hyperthyroidism (IIH). Historically, this phenomenon was mainly suffered by I-deficient patients exposed to increased amounts of I. Currently, the problem affects patients with pre-existing thyroid disease (including multinodular goiter and Graves’ disease goiter) who have been treated with I contrast agents or amiodarone. These individuals have impaired or absent autoregulatory mechanisms that reduce thyroid hormone production. JBP usually resolves spontaneously after I exposure ceases within a few weeks or months [133,134]. A higher incidence of Graves’ disease, particularly in young people, is also linked to higher dietary I levels. Since I shortage is just as detrimental, adequate doses of I supplementation should be sought [135,136,137,138]. More research is required to pinpoint the precise mechanism by which excess iodine contributes to the onset of Graves’ disease. I is used in radioactive form to treat Graves’ illness, even though it influences its progression. In the course of Graves’ disease, I-131 can be administered as the primary therapy for hyperthyroidism. The efficacy of radiotherapy after less than 2 months of treatment was 53.7%, and after 12 months of follow-up, remission was achieved in 88.88% of patients. A high single dose of radioactive I provides a high remission rate but is correlated to an elevated severity of hypothyroidism [139]. Following radioiodine therapy, there was no difference in the development of overt primary hypothyroidism between patients with and without reduced I consumption. Although the group of patients with reduced I intake showed trends toward improved treatment results six months following radioiodine treatment, the difference was not statistically significant [140]. An increased risk of radioactive I (RAI) failure is seen in patients with increased thyroid volume, longer isthmus length (cutoff value 5.2 mm), higher 131 I uptake rate, and longer duration and higher dosage of anti-thyroid drugs (ATD) [141,142]. Conversely, patients with smaller thyroid masses and lower FT4 levels had superior results [143].

#### 3.4.2. Iodine and Hashimoto’s Thyroiditis

An autoimmune condition called Hashimoto’s thyroiditis may be brought on by intake of too much I. In vitro, high I causes increased pyroptosis activity in thyroid follicular cells in the thyroid tissues of people with Hashimoto’s thyroiditis. The ROS-nuclear factor kappa-light-chain-enhancer of activated B cells (NF-κB) signalling pathway and activation of the nucleotide-binding domain, leucine-rich–containing family, pyrin domain–containing-3 (*NLRP3*) inflammasome are involved in excessive pyroptosis, which in turn leads to the release of interleukin 1β (IL-1β) by the *NLRP3* inflammasome [144]. Other studies have shown that excess I promotes FTC apoptosis by activating the hypoxia-inducible factor 1alpha (*HIF-1α*)-mediated hypoxia signaling pathway and increasing *N*-myc downstream regulated 1 (*NDRG1*) expression and inhibits FTC proliferation in a dose-dependent manner [145]. Furthermore, too much iodine can reduce autophagy activity in thyroid follicular cells and increase ROS generation by downregulating transforming growth factor β (TGF-β) and activation of the *AKT*/mammalian target of rapamycin (*mTOR*) signaling pathway [146]. It has been discovered that giving mice moderately high doses of iodine causes naive T cells in the spleen to polarize into Th17 cells, whereas giving them extremely high doses of iodine enhances Th1 polarization and prevents the production of regulatory t cells (Treg) [147]. I supplementation during pregnancy does not increase TPOAb and does not adversely affect the course of Hashimoto’s disease, so it can be used [148]. Elevated urinary I levels are correlated with an elevated risk of Hashimoto’s disease. Furthermore, it proved that a more than twofold increase in the likelihood of hypothyroidism—Hashimoto’s thyroiditis—was linked to an increase in urine I [149].

#### 3.4.3. Iodine and Hypothyroidism

Excess I can result in hypothyroidism and the development of the WCE in addition to the I-based phenomena. It is most likely connected to the inhibition of TPO, which lowers Tg iodination, as a result of the synthesis of inhibitory chemicals (intrathyroidal iodolactones, iodoaldehydes, and/or iodolipids) [150,151]. This phenomenon occurs within 48 h to 10 weeks after acute I excess and resolves within 2 days to 5 months as a result of the escape mechanism [152]. It involves a reduction in the activity of surface sodium I symporters, resulting in lower intrathyroidal I levels. As a result, the amounts of chemicals that prevent the synthesis of TH drop, and normal TH production resumes. [150,151]. The runaway phenomenon may not occur in the absence of cessation of I exposure and immature filtering function of the kidneys, which is particularly important for newborns. When radiological examinations are performed with an I contrast agent, the incidence of developing hypothyroidism reaches 8.3% of term newborns and 18.3% of preterm infants and is the most common in children under 4 years of age at <3 months [151,153]. Exposure to I contrast media (ICM) also increases the risk of hypothyroidism among children and adolescents [154]. The risk of developing hypothyroidism is increased in cases of monoallelic dual oxidase 2 (*DUOX2*) variant, hypothyroidism, or preterm birth [155]. Lack of adaptation to WCE can also appear in patients with autoimmune thyroiditis, treated with radioactive I and antithyroid drugs for Graves’ disease, postpartum thyroiditis, subacute thyroiditis, amiodarone-induced thyrotoxicosis type 2, hemithyroidectomy or interferon-α (IFNα) therapy [150].

I contrast media can induce thyroid dysfunction in the form of both hypothyroidism and hyperthyroidism. The disorders appear within 30 days of ICM application, most commonly within the first 10 days [156]. According to a US study, hypothyroidism with frequent I contrast delivery was linked to an 11% higher risk of heart failure over a mean follow-up period of 3.6 years, with the risk being higher for women [157]. Another study shows a 1.17-fold increased risk of thyroid disease (especially hypothyroidism) among patients with ICM exposure at the time of computed tomography (CT), compared to patients without ICM exposure [158]. Therefore, it is important to monitor thyroid function during this period [156]. There are conflicting studies on I deficiency in children fed parenterally. In Brazil, a series of cases of hypothyroidism in parenterally fed children were observed, which was associated with I deficiency due to a lack of I supplementation in parenteral nutrition solutions. A similar problem occurs in children with intestinal diseases in which malabsorption is impaired [159]. In a different study assessing the issue of iodine deficit in infants receiving parenteral nutrition, feeding for more than 15 days was linked to higher serum TSH levels in 48.9% of cases, while hypothyroidism was present in 10.5% of cases overall [160]. A study on a group of 15 children aged 1–17 years showed I deficiency despite I supplementation of 1 μg/kg/day [161]. In contrast, a study on a group of 32 children aged 6 months to 18 years found no patients with I deficiency or thyroid dysfunction [162]. Hypothyroidism can also occur in children who are I deficient and fed soy-based infant formula due to cow’s milk allergy or lactose intolerance. TPO is inhibited by the phytoestrogenic isoflavonoids in soy, which serve as substitute iodination substrates. I deficit makes this process worse [163]. I absorption is also impaired among children with celiac disease, where I deficiency was found in 80.9% of subjects at baseline [164]. Perchlorates cause a decrease in I absorption, but it appears that their concentrations in drinking water are low and do not affect normal thyroid function [165]. Perchlorate levels in water and drinking water and TSH levels in umbilical cord blood have also been investigated, but no differences in TSH levels have been shown depending on perchlorate concentrations in drinking water in the region [166]. A study in Nepal showed that reduced I levels in children elevated thyroid autoimmunity risk. There was an increase in TgAb levels in this group, but TPOAb was not assessed, and the study group was not large [132]. In Nepal, 34.4% had excessive urinary I excretion indicative of excessive dietary supply [167]. Pregnant women who take too much I run the risk of giving birth to a baby with temporary or permanent hyperthyrotropinemia, which could have a negative impact on the child’s neurodevelopment if left untreated. It has been reported in the case report that daily seaweed ingestion by the mother during pregnancy and lactation can result in temporary congenital hypothyroidism [168]. Children born with congenital hypothyroidism did not have statistically significantly elevated or decreased I levels, but higher blood I concentrations were found in infants treated in the neonatal intensive care unit (NICU), indicating overexposure to I during treatment and the need to monitor I exposure during surgical procedures, imaging studies, and the use of I disinfectants [169]. Additionally, infants are more susceptible to iodine deficit: in regions with moderate to severe iodine deficiency, the incidence of hypothyroidism in early infants is four times higher than in groups of mothers [170].

#### 3.4.4. Iodine and Autoimmune Thyroiditis

The development of autoimmune thyroiditis is influenced by numerous risk factors, making it a multifactorial disease. One such factor is I excess. Where I supplementation in the form of salt iodization has been implemented, an increase in the prevalence of autoimmune thyroiditis has been noted [171]. Elevated urinary I excretion (UIE) was observed in the group of children with autoimmune thyroiditis compared to the healthy control group [172]. Excessive I levels can affect a variety of changes in the expression of various genes involved in the pathogenesis of autoimmune thyroiditis. It has been shown that patients covering >90% of their I requirements with qualified iodized salt have higher methylation of tyrosine 3-monooxygenase/tryptophan 5-monooxygenase (*YWHAG*) and brain-selective kinase 2 (*BRSK2*) with lower methylation of inhibitor of growth 4 (*ING4*) and interact with methylation levels of these genes, resulting in an increased risk of developing autoimmune thyroiditis [173]. The study from 2023 showed that autoimmune thyroiditis is correlated to hypomethylation of Ras-related protein Rab-8A (*RAB8A*) and Ras-related protein Rap-1A (*RAP1A*), which corresponded to higher levels of mRNA expression. Patients with increased I exposure experience hypomethylation of most of the CpG locus of *RAB8A*, olfactory receptor family 4 subfamily K member 17 (*OR4K17*), and *RAP1A*, indicating that excess I is involved in the pathogenesis of autoimmune thyroiditis [174]. I supplementation after long-term I deficiency, as well as I excess, can affect the DNA methylation levels of protein kinase AMP-activated catalytic subunit alpha 2 (*PRKAA2*) and integrin subunit alpha 6 genes (*ITGA6*), with mRNA expression of the *ITGA6* gene negatively correlated with DNA methylation levels of this gene [175]. Another study evaluated DNA methylation of genes related to the extrinsic apoptosis signaling pathway in autoimmune thyroiditis in areas with different I levels; an increase in death-associated protein kinase 1 (*DAPK1*) DNA methylation was observed in autoimmune thyroiditis patients, and a concentration-dependent effect of I on *DAPK1* and tumor necrosis factor alpha-induced protein 8 (*TNFAIP8*) gene methylation was noted [176]. Different I content in water is also associated with different methylation patterns of major histocompatibility complex, class II, DP beta 1 (*HLA-DPB1*), and programmed cell death 1 ligand 2 (*PDCD1LG2*) genes related to the cell adhesion molecule pathway, which may play a role in the pathogenesis of autoimmune thyroiditis [177]. More research is required to determine how various biological processes contribute to the onset of autoimmune thyroiditis.

In addition to epigenetic changes, excess I also affects the immune system, potentially causing or exacerbating autoimmune thyroiditis. A study in mice showed that excess I with prolonged exposure causes infiltration of the thyroid gland by Th9 cells, especially near damaged thyroid follicular cells, and increased expression of IL-9 mRNA and protein and key Th9 cell transcription factors (PU.1 and IRF-4). In addition, an increase in TgAb and IL-9 antibodies and enlargement of the spleen were observed. The greatest severity of the changes was observed after 8 weeks of continuous exposure to elevated I levels and correlated with the greatest severity of the disease. To ascertain Th9’s function in the pathophysiology of autoimmune thyroiditis, more research is required [178]. Th1 and Th17 cells are important subsets of Teff in the pathophysiology of spontaneous autoimmune thyroiditis, according to another study in mice that revealed higher numbers of Th1 and Th17 cells in the spleen and accumulation of both types of Teff in the thyroid gland of I-fed wild-type mice [179]. In another study on non-obese mice with H2 h4 diabetes (NOD. H-2 h4) induced by excess I, an increase in the incidence of autoimmune thyroiditis, the number of Th17 cells, and the expression of IL-23, IL-17, IL-6, and interferon-γ (INF γ) (and a decrease in the protein and mRNA expression of IL-4 and interferonγ) were observed in the group of mice receiving sodium iodide (NaI). In the autoimmune thyroiditis mice, the expressions of retinoic acid-related orphan receptor gamma t (*RORγt*), retinoic acid-related orphan receptor alpha (*RORα*), and signal transducer and activator of transcription 3 (*STAT3*) were much higher, but the expression of forkhead/winged helix transcription factor p3 (*Foxp3*) is significantly lower [180]. I interacts synergistically with IFN-γ to increase *ICAM-1* expression on thyrocytes in a mouse model of thyroiditis [181]. Furthermore, fewer clusters of differentiation 4 (CD4) + CD25 + Foxp3 + T cells were observed in mice with I-induced inflammation, and this decrease increased with prolonged I treatment [175]. Iodide-induced thyroid follicular cells are stimulated to secrete chemokines such as CCL2, CXC motif chemokine ligand 8 (CXCL8), and C-X-C motif chemokine ligand 14 (CXCL14), which together with intercellular adhesion molecule-1 (*ICAM1*) attract immunocompetent cells to the thyroid gland [182]. In addition, I enhances the antigenicity of Tg and increases the reactivity of TgAb and some lymphocytes in NOD.H-2 h4 mice, and excess I causes increased inflammation, apoptosis, and DNA damage to follicle epithelial cells of mouse thyroid, accompanied by reduced targeting MutT homolog 1 (*MTH1*) expression [183,184].

#### 3.4.5. Iodine and Thyroid Nodules

Thyroid nodules are prevalent, particularly in areas with deficient I levels [185]. I shortage results in decreased TH production, which impairs the HPT axis’s negative feedback mechanisms and increases the secretion of thyroid-stimulating hormone, which in turn causes the thyroid gland to be continuously stimulated. In I-deficient populations, it results in an increased incidence of thyroid illness, goiter, thyroid nodules, and PTC [186]. However, despite normal I levels, the incidence of thyroid nodules is high [187]. Because of USI, the incidence of goiter decreased in China after 1996, while after 2002, the incidence of thyroid nodules and goiter significantly increased [188]. One study showed a reduction in thyroid nodule risk in men with increasing urinary I concentration (UIC) when UIC was less than 527 μg/L, while among women, UIC and the presence of thyroid nodules were negatively associated [189]. Another publication indicates that the relationship between UIC and thyroid nodule risk is U-shaped, with consumption of non-iodized salt being an independent risk factor for thyroid nodules [190]. It has also been shown that especially in individuals at high risk for malignant tumors, failure to adhere to the Mediterranean diet was linked with nodular thyroid disease [191]. Treatment can include I-131, which is a well-established therapeutic option with a high success rate [192,193].

#### 3.4.6. Iodine and Thyroid Cancer

It is unclear if I contributes positively or negatively to the development and spread of thyroid cancer in terms of risk assessment [194]. In South Korea, patients under 45 years old who had greater creatinine-adjusted UIC had a linearly increased risk of PTC [195]. An elevated PTC risk was also reported as a result of near-daily seaweed consumption in postmenopausal women [196]. More than 5 g of iodized salt per day was found to raise the incidence of thyroid cancer and thyroid nodules [197]. In contrast, a meta-analysis found that excessive I intake, i.e., UIC 300 ug/L, was linked with the prevalence of PTC, while adequate intake of I (100 ≤ UIC < 200 ug/L) could be one of PTC’s protective factors [198]. An analysis of thyroid cancer incidence in China following USI revealed a decrease in the incidence of FTC and an increase in the detection of PTC and MTC, but no change in the incidence of undifferentiated thyroid cancer (UTC). Furthermore, the incidence of PTC exacerbated by nodular goiter or chronic lymphocytic thyroiditis rose, while the mean age of patients with thyroid cancer decreased [199]. Similar changes were observed in Austria where there was also an elevation in the incidence of thyroid cancer with a predominance of PTC (54.4%) and a decrease in anaplastic thyroid cysts [200]. In Slovenia, a decrease in thyroid carcinoma was also observed as a result of salt iodization. While the number of distant metastases has remained constant, patients were less likely to suffer lymph node metastasis [201]. Studies are also available in which patients with increased dietary I intake were observed to have a reduced risk of PTC, with a slight rise in PTC risk in the “high-risk” women group. According to the authors, I exposure is associated with at most a poor risk of PTC [202]. The results of a 2017 meta-analysis indicate a protective effect of higher I intake (≥300 μg/d) for the development of thyroid cancer [203]. This supports findings from another study that found inadequate iodine intake to be a risk factor for thyroid cancer [204]. Another study indicated that the protective effect of I may be enhanced by the solute carrier family 5 member 5 (*SLC5A5*) rs77277498 genotypes [205]. Low I intake is a protective factor for central lymph nodes (CLNM) in PTC with tumor diameter < 1 cm [206]. Seaweed consumption may be a protective factor against the development of thyroid cancer, while dairy products may be helpful in lowering the incidence of thyroid cancer. These findings were obtained when examining the impact of iodine-rich foods on the risk of developing thyroid cancer. However, due to the limitations of the study due to the fact that only questionnaires were conducted, without histological studies or determination of urinary I levels, more research is required to ascertain this link [207].

Regarding the impact of I levels on the prevalence of *BRAF* mutations, there are variations. More authors indicate that there are no differences in *BRAF* mutations depending on I levels [198,208,209]. In contrast, one study showed that low or excessive I intake can be found as a risk factor for *BRAF* mutations in PTC in I-rich areas. The mutation rate was lowest in the group receiving UIC 300–499 µg/L [210]. *BRAF* mutations were found to be significantly associated with lymph node metastasis, extrathyroidal invasion, and advanced PTC tumor stage, and they were more common in iodine-rich areas [211]. Whereas, a study conducted on rats found a protective effect of I by inhibiting *BRAF* V600E-induced activation of the neurogenic locus notch homolog protein/microRNA 19a (Notch/miR-19) loop and restoring sensitivity to TGF-β signaling [212]. In regions with low I levels, a rise in the prevalence of rat sarcoma virus (*RAS*) mutations has been observed and thus influences the initiation and/or maintenance of follicular tumors. However, the authors indicate that additional factors are required to initiate carcinogenesis [213]. In contrast, another study found no differences in the incidence of rearranged during transfection (RET)/PTC or *RAS* mutations between I-rich and I-poor areas [214]. An in vitro study showed different effects of I on thyroid cells depending on concentration. I at low concentrations activated the *AKT* and *ERK* signaling pathway in thyroid cancer cells and caused an increase in the number of migrating thyroid cancer cells. In contrast, I at high concentrations inhibited the *AKT* and *ERK* signaling pathway and caused a decrease in the number of migrating thyroid cancer cells. I concentrations below 1.0 × 10^−3^ mM were found to promote thyroid cancer cell growth and migration, whereas higher concentrations of I inhibited these processes. In addition, the same study showed a link between I and the secretion of vascular endothelial growth factor (VEGF-A), the expression of which correlated with microvessel density (MVD) and poor prognosis of human cancers [194]. Exposure to large amounts of I increased the activation of AKT phosphorylation and the expression of phospho-Wee1 (Ser642) while decreasing the expression of phospho-cyclin dependent kinase 1 (*CDK1*) (Tyr15). These changes induce the proliferation of thyroid cancer cells [215]. In a study from 2016, which was conducted on I-exposed cells, higher expression of sperm protein associated with the nucleus, X-linked, family member A1 (*SPANXA1*) was observed, while I treatment resulted in increased expression of *PI3K* and *p-AKT*. As a result, PI3K/AKT might be one of the main signaling pathways through which I promotes the development of thyroid cancer in association with *SPANXA1* [216]. It was shown that at high levels of I, the frequency of autophagosomes and autophagosome-lysosomes in human PTC cells elevated significantly, as did 1A/1B-light chain 3—phosphatidylethanolamine conjugate (LC3-II) and *BRAF* kinase activity. High levels of I promoted apoptosis and migration of PTC cells and inhibited cell proliferation. At high I concentrations, autophagy triggered by *BRAF* kinase improved proliferation and migration while also preventing apoptosis [217].

Thyroid cancer can be treated with radioactive iodine, although iodine-131 therapy is ineffective in patients who are radioiodine resistant (RAIR). An increase in tartrate and shikimic acid, (metabolites of the tyrosine pathway) and a decrease in hippuric acid and 2-phenylacetamide (metabolites of the phenylalanine pathway) were observed in this patient group. These changes in metabolic pathways are closely linked to the development of I resistance and could be the reason why radioiodine fails [218]. The development of I resistance may be associated with *BRAF* and/or telomerase reverse transcriptase (*TERT*) promoter mutations and to a lesser extent tumor protein p53 (*TP53*) and dicer 1 ribonuclease III (*DICER1*) mutations [219,220]. In preclinical studies, it has been shown that the development of resistance may be related to *ALK* (anaplastic lymphoma kinase) fusion-induced downregulation of NIS. Inhibiting downstream signaling pathways or *ALK* can reverse this condition [221]. In I-refractory differentiated thyroid cancer (RAIR-DTC) patients, 125I seed implantation may be a better treatment option. Patients treated with this technique have reported less pain and tumor size reduction [222]. Quantitative parameters such as SUVmax, SUVmean, and %ID obtained from I-131 single photon emission computed tomography (SPECT)/CT imaging of the thyroid bed, which were positively correlated with stimulated thyroglobulin (sTg) and inhibitory Tg at 6 months after RAI adjunctive therapy, respectively, can be used to assess potential outcomes of RAI adjunctive therapy [223]. Furthermore, the uptake of 99 m-nadtechnetate Tc can be evaluated in DTC patients with lymph node metastases. A greater benefit of 131 I therapy has been noted in patients sensitive to 99 m Tc-supertechnetate [224]. Because high levels of Tg and TgAb are linked to worse treatment response, greater relapse rates, higher cumulative doses, and more I intake, studies suggest that measuring baseline Tg and TgAb levels may be beneficial. When TgAb and Tg levels are elevated, the authors advise careful monitoring until Tg and TgAb levels return to normal [225]. In addition, Tg levels alone correlate with I-131 uptake and retention [226].

#### 3.4.7. Iodine and Postpartum Thyroiditis

Postpartum thyroiditis is a condition that occurs up to 1 year after delivery and usually manifests 1–4 months after delivery. Because of its unique treatment, it needs to be distinguished from other disorders of the thyroid [227]. The risk of postpartum thyroiditis and its severity, as determined by the length of time and degree of deviation of TSH and thyroid hormone levels from normal, are unaffected by I supplementation during pregnancy or the postpartum phase. The level of TPOAb, which can be measured in the early stages of pregnancy to determine the risk of postpartum thyroiditis, is the primary determinant of the incidence, severity, and type of postpartum thyroiditis [228,229,230].

### 3.5. Manganese

Mn is one of the key trace elements for mammalian function. It has low mutagenic or carcinogenic properties. Mn builds a number of enzymes engaged in defense against ROS: oxidoreductases, Mn-SOD, lyases, isomerases, transferases, hydrolases, ligases, and glutamine synthetase. Mn-SOD plays a key role as an enzyme in metabolizing superoxide anions. This lessens the damage that free radicals cause [231]. Importantly, the findings suggest that Mn can interfere with deiodinase activity, so it can affect the concentration of circulating thyroid ions [39].

#### 3.5.1. Manganese and Hypothyroidism

Treatment with excessive amounts of Mn can cause enlargement of the thyroid gland, as has occurred in female mice [232]. Compared to healthy individuals, patients with Hashimoto’s thyroiditis had greater serum and thyroid tissue Mn concentrations, which can also reduce the levels of FT3 and FT4, leading to hypothyroidism [2]. Studies are proving elevated Mn levels in autoimmune hypothyroidism. It could therefore be an indicator of the risk or degree of thyroid damage [27]. According to a different study, hypothyroidism is inversely correlated with the consumption of antioxidant minerals like Mn. This implies that oxidative stress may be linked to hypothyroidism [233]. Another study shows that Mn levels in women’s blood and TSH are negatively correlated. Additionally, Mn levels were higher in patients with multinodular goiter than in healthy subjects [11].

#### 3.5.2. Manganese and Thyroid Cancer

In the case of thyroid cancer, researchers have found that patients have higher blood Mn levels [234]. In the single-element models, Mn has also demonstrated a strong correlation with thyroid tumors [235]. This can be used as an indicator of tumor development and progression. In another study, patients with thyroid cancer were found to have increased amounts of Mn in thyroid tissues compared to patients with benign thyroid disease. No such changes were noted in the blood. This may be due to increased levels of Mn-SOD in tumor tissue. However, this study shows how limited knowledge is about heavy metals and their effects on the human body [236]. Another study shows a tendency to reduce Mn levels in tissues affected by MTC [106]. According to a study on how Mn compounds affect thyroid cancer, MnO_2_/CDDP@PDA-Cy5.5 can efficiently eliminate cancer cells in vitro. However, in vivo research has demonstrated that the drug delivery system can supply medications to the tumor location steadily, which will greatly slow the growth of the tumor. Additionally, once the drug delivery mechanism has degraded to Mn^2+^ at the tumor site, it can be used for MRI [237]. Consequently, it is possible to attain the dual advantages of tumor diagnostics and treatment. Another study involving the formation of HSA-MnO_2_-131I enabled imaging and treatment of ATC. To enhance the therapeutic impact, it reduced the diffusion of radionuclides and maximized accumulation in the tumor area. At this point, the state of our knowledge about Mn in the human body is limited, but hopefully, the future will bring us a solution to many unknowns [238].

### 3.6. Zinc

Zn is one of the trace elements that significantly affects thyroid function. We can find it in products such as pumpkin seeds, flax seeds, and whole grain cereal products [60]. Zn is part of antioxidant enzymes, as well as a transcription factor that binds TH [239]. In turn, a lack of Zn affects the production of TRH, TSH, T3, and T4 [240]. The relationship between Zn and thyroid function appears to be mutual. Insufficient Zn supplementation causes hypothyroidism, while hypothyroidism can lead to Zn deficiency [49]. In chronic and autoimmune disorders, Zn deficiency can impair immune function and cause the generation of pro-inflammatory cytokines [50]. Therefore, it may serve as an element of immune system control in the future.

#### 3.6.1. Zinc and Hashimoto’s Thyroiditis

As for studies conducted on the blood serum of females with Hashimoto’s thyroiditis, they showed no relationship between Zn levels among patients and controls [83]. Rather, another study indicates that normal thyroid function is restored in individuals with Hashimoto’s disease when Zn supplementation returns to normal levels. In the treatment and diagnosis of Hashimoto’s disease, focus ought to be given to concomitant diseases that can lead to malabsorption, which can result in a deficiency of elements such as Zn [60]. Oxidative stress and apoptosis play a main role in the etiopathogenesis of Hashimoto’s thyroiditis, so maintaining the right balance can help improve thyroid function. Numerous studies show that Zn is essential to this process [241].

#### 3.6.2. Zinc and Hypothyroidism

The effects of Zn on thyroid function in hypothyroid patients were investigated in a randomized, controlled study, which showed that Zn supplementation significantly reduces serum hs-CRP (high-sensitivity *C*-reactive protein) levels and significantly increases serum FT4 levels [14]. Dietary Zn intake lowers the risk of hypothyroidism, according to another study. Low dietary Zn consumption may contribute to the development of hypothyroidism, which is why it is important to control Zn intake in overweight individuals and pregnant women [242]. Another study’s findings show that hypothyroid patients’ serum Zn levels were considerably lower than those of the control group. Furthermore, there was a negative correlation between Zn and T3, suggesting a strong link between Zn and hypothyroidism [243].

#### 3.6.3. Zinc and Autoimmune Thyroiditis

According to a different study, serum Zn levels were lower in patients with autoimmune thyroiditis and hypothyroidism than in healthy individuals. However, no correlation was found between Zn deficiency and the type of thyroid disease [90].

#### 3.6.4. Zinc and Thyroid Nodules

According to research on the microscopic calcification of thyroid nodules, Zn in fine needle aspiration specimens may serve as a malignancy biomarker for a more precise diagnosis [244]. Interestingly, no such relationship was detected in men, meaning that the results were gender specific. Additionally, only in women was a positive correlation found between elevated metal levels and cancer mortality. Urinary Zn levels were lower in nodular goiter patients than in controls, according to a case-control study, and were inversely correlated with the likelihood of nodular goiter development [93]. An association between Zn levels and thyroid nodules was shown in yet another study, where those with high Zn levels were at higher risk for thyroid nodules [69]. Another study, in turn, suggests that Zn deficiency may contribute to thyroid nodular goiter and may also be correlated with the formation of thyroid nodules [94].

#### 3.6.5. Zinc and Thyroid Cancer

Zn fingers (ZHX2), or transcription factors, play an important role in thyroid cancer development. Moreover, they may be engaged in its metastasis. One study indicates that ZHX2 inhibits thyroid cancer metastasis by inhibiting calcium-binding protein expression at the transcriptional level [245]. Researchers have also focused on another Zn finger protein (*ZNF703*). They observed a relationship between its expression and the pathological stage of MTC, tumor size, and lymph node metastases. Thus, it is possible that *ZNF703* is a therapeutic target, because it may play the role of a crucial regulator of MTC growth and progression [246]. One study tells us that all cancers are Zn-deficient lesions, so raising Zn levels is a major treatment target in the form of Zn chemotherapy. Thus, the conclusion can be drawn that “Zn is the wonder drug for the treatment of carcinomas” [247]. Unfortunately, not every study supports this. Some show a lack of an association between Zn supplementation and thyroid cancer prevention [72]. Rather, a different study reveals that PTC patients had higher Zn levels than healthy people, which could be important in the future as further biomarkers for thyroid cancer identification. The state of current knowledge allows us to draw some conclusions, but there is still much to be discovered. Hopefully, in the future, we will learn more details about the effects of Zn on the functioning of the human body [108].

### 3.7. Silver

Ag is an element that has long been used in healthcare. It owes its popularity in particular to its antimicrobial action due to its active Ag+ ion, which interacts with DNA, leads to the disturbance of the cell wall and/or membrane, inhibits proteins and enzymes of bacteria, and, within the bacteria, leads to the production of ROS. It is also used to prevent bacterial infections, e.g., in burns, but also in wound dressing. AgNPs are also currently used. Thanks to advances in nanotechnology, they can be used together with antibiotics to reduce their dosage or even replace antibiotics in the treatment of infections with antibiotic-resistant strains. However, studies also show the existence of Ag-resistant strains [248,249]. As the toxicity of Ag is low, inhaling it, swallowing it, or applying it to the skin is associated with little clinical risk. The only adverse consequence of prolonged Ag ingestion is its accumulation in organs, including the skin and eye, but this is not life-threatening and only affects the cosmetic effect. After absorption as a protein complex, Ag enters the circulatory system and is eliminated by the liver and kidneys. Its metabolism is regulated by the metallothioneins (MT) with which it binds. A contraindication to the use of Ag is the presence of allergies [250].

#### Silver and Thyroid Cancer

In the case of thyroid tumors and cancers, Ag is an interesting element: due to its possible involvement in their formation, on the other hand, Ag nanoparticles are being tested as a possible anticancer drug. In a study carried out on 135 patients with various thyroid tumors, the concentration of trace elements, including Ag, in the tumors and apparently intact paranodular thyroid tissue was analyzed. It was proven that compared to the normal thyroid of healthy people, the Ag level was higher in each case, benign nodules, malignant tumors, and paranodular tissue, in which more Ag was detected in the case of a malignant lesion than in the case of a benign lesion. Moreover, in malignant tumors themselves, the Ag concentration was lower compared to benign tumors. There are homeostasis mechanisms in a healthy thyroid that keep the amount of Ag and other elements at a constant level. However, in the case of their increased accumulation, which exceeds the defense capabilities, toxic effects on thyrocytes occur. This could contribute to the development of thyroid cancer [126]. However, in the study of AgNPs obtained from Livistona chinensis leaf extract (LC-AgNPs), which was performed on human PTC cells (TPC1 cell line), their anticancer effect was demonstrated. Following the application of free AgNPs and LC-AgNPs, a reduction in the number of cells from the TPC1 cell line was noted. The decrease in cell number was significantly larger in the case of LC-AgNPs. Although the exact mechanism has not been discovered, due to the presence of phenolic compounds, induction of apoptosis has been suggested [251]. Another study confirming this anticancer property of AgNPs used AgNPs synthesized by Pseudomonas aeruginosa (Ps-AgNPs). They were also applied to the TPC1 cell line, and it was shown that ROS induced apoptosis and inhibited cell proliferation. Overproduction of these can damage mitochondria and DNA, which ultimately results in cell death. The damage and ROS production increased with increasing doses of Ps-AgNPs [252]. Another example of the effect on PTC is the tested gold and silver alloy nanoparticles coated with polydopamine (Au–Ag@PDA NP). These molecules can accumulate in mitochondria and cause their dysfunction. Cell proliferation is then inhibited when the cell cycle reaches the S phase and the p53 level rises. This mechanism enables non-invasive treatment of PTC [253].

### 3.8. Cadmium

Cd is one of the elements classified as heavy metals, which is characterized by significant toxicity. The sources of infection for exposed people are cigarette smoke and the work environment, while for everyone, it is due to contaminated food and beverages and diet. The main foods containing Cd are mollusks, crustaceans, oilseeds (sunflower seeds, peanuts, linseed), and organ meats. There are several diseases and organ dysfunctions that can result from Cd exposure, such as kidney damage, osteoporosis, diabetes, hypertension, and lung diseases. Cd is also a carcinogen. Exposure at work is linked to lung cancer, but Cd can also cause prostate and kidney cancer. The absorption of Cd from the lungs is very high and reaches over 90% of the dose compared to the oral route, where only 5% is absorbed. However, regardless of the absorption route, rapid blood removal causes Cd to build up in tissues, primarily the liver and kidneys [254,255]. The protein MT is responsible for the accumulation of Cd in organs and for ensuring that the metal has a lengthy biological half-life in the body [256]. The thyroid is an organ where Cd can also accumulate throughout life. High concentrations are observed after the age of 20, and are the highest in the age group 40–60 [257]. This accumulation of the element may lead to various thyroid dysfunctions.

#### 3.8.1. Cadmium and Hypothyroidism

Cd significantly affects thyroid function by reducing the level of TH produced, so Cd exposure is associated with a tendency to develop hypothyroidism, as confirmed by a Korean nationwide cross-sectional study. The value of FT4 decreased with the increase in Cd blood level; interestingly, this relationship was more noticeable in men [258]. In a rat study, this effect of Cd on T4 was previously shown. Serum T4 levels were lower in rats given Cd than in control rats. Rats exposed to Cd also showed noticeably elevated TSH levels [259]. Similar results were seen in rabbits, where compared to the control group, TSH levels increased, and average T3 and T4 values decreased [260]. In the Korean study, no correlation between TSH levels and Cd was observed. However, it was proven that the level of Cd in the blood, although there was no significant correlation with TPOAb, was observed at the highest level of TPOAb at the highest tested level of Cd [258]. Another study conducted on a group of workers to detect the impact of occupational exposition to Cd on thyroid function showed significantly higher serum TPOAb concentrations in the exposed group compared to the control group. Additionally, workers had greater TSH levels than those who were not exposed. Researchers came to the conclusion that autoimmune hypothyroidism and Cd exposure are related [8]. In a study drawing data from two cross-sectional studies, high Cd concentrations in the blood were correlated with the presence of TPOAb antithyroid antibodies. However, in contrast to the earlier study, there was no discernible link between Cd exposure and the thyroid gland’s hormone-secreting activity [261]. A total of 110 people were also examined to assess the level of trace metals in serum, including Cd, in people with thyroid diseases, including hypothyroidism, as well as in healthy people. It turned out that the serum Cd level was higher in sick people compared to healthy people. Increasing Cd levels have also been found to increase the risk of hypothyroidism [88]. A study conducted in China also indicates a relationship between Cd levels in the blood and hypothyroidism, and this positive association was particularly noticeable in women. High levels of TgAb were also observed with high Cd concentrations in both sexes [262].

The malfunctioning action of Cd on thyroid follicles may be the cause of the thyroid gland’s decreased hormone output when under its influence. In a study on mice’s thyroid glands, it was found that Cd raised the height of the follicular epithelium, decreased the surface area of follicles, and nearly tripled the amount of perifollicular connective tissue in comparison to the control group. Furthermore, it was demonstrated that Cd stimulates the production of monocyte chemoattractant protein-1 (MCP-1) and the CXC motif chemokine 10 (CXCL10), as cells expressing MCP-1 and CXCL10 lined the follicle walls following Cd administration. Chemokines MCP-1 and CXCL10 play a role in the development and upkeep of Hashimoto’s thyroiditis [263]. In another study on thyroid tissues of adult male mice in which Cd caused a significant reduction in levels of T4 and T3 when observed under a microscope, significant growth in epithelial cell height was observed, consistent with the previous study of thyroid follicles, as well as their enlargement and hyperplasticity. The surface area of the thyroid follicles decreased, and the follicles had irregular shapes. By triggering the ERK1/2 pathway, creating endoplasmic reticulum stress, and generating ROS generation and lipid peroxidation, the Cd also induces structural alterations in the nucleus, mitochondria, and endoplasmic reticulum in alveolar epithelial cells as well as apoptosis of these cells [264,265]. Additionally, it was discovered that Cd, apart from structural changes and promoting apoptosis of thyroid follicular cells, also facilitates the infiltration of macrophages and causes an inflammatory reaction in the thyroid gland. Inflammatory cytokines such as IL-1β, IL-6, and tumor necrosis factor-alpha (TNF-α) were produced in greater quantities when Cd was present. In addition to apoptosis, it has been proven that Cd can also lead to pyroptosis of thyroid cells via NLRP3 and by regulating the nuclear factor erythroid 2–related factor 2/Kelch-like ECH-associated protein/nuclear factor kappa-light-chain-enhancer of activated B cells (*Nrf2/Keap1/NF-κB*) pathway, which leads to impaired thyroid hormonal function [266]. On the other hand, in a study from the US, blood Cd was linked to a low TSH level, whereas T3 or T4 levels were within the normal range, which could indicate subclinical primary hyperthyroidism. However, when measuring metals in urine, Cd was correlated with higher concentrations of T3 and T4, and the TSH level was unchanged, which rather suggests secondary hyperthyroidism. There are some differences and discrepancies in the studies. This suggests that this issue requires further research [267].

#### 3.8.2. Cadmium and Thyroid Cancer

Cd is considered a carcinogen, which disrupts both the function and structure of the thyroid gland. Cd damages the structure of follicular cells and C cells. It leads to diffuse hyperplasia, as well as nodular hyperplasia of C cells and C cell microadenomas, which are pre-cancerous lesions [263,264,265,268]. A study on the thyroid glands of rats administered Cd also showed morphological pre-cancerous changes caused by this element. Diffuse colloidal goiter occurred most frequently. The follicular hyperplasia contained cystic follicles filled with colloids and covered with single-layered, low-volume atrophic thyrocytes. Microvesicular hyperplasia was also common [269]. An increased thyroid cancer incidence, mainly papillary histotype, was observed in people from volcanic areas. In this area, the values of trace elements, including Cd, were measured in both drinking water and lichens, confirming water and air pollution. In contrast to the control group, this level was higher. Residents of the volcanic zone also had higher levels of Cd in their urine. This may suggest that Cd is one of the factors influencing thyroid cancer occurrence [270]. Compared to healthy tissue, papillary thyroid tissue in PTC patients had significantly higher Cd levels. Thyroid growth in the retrosternum was likewise impacted by Cd [271]. In a study conducted on Korean women with various stages of PTC, the concentrations of heavy metals and trace elements, including Cd, in blood and thyroid tissue were examined. There was a positive correlation between the levels of Cd in tissues and blood. Additionally, compared to patients with a T1 stage of cancer, those with a ≥T2 had higher amounts of Cd in their tissues. Thus, it appears that Cd could be a contributing component to the development and exacerbation of thyroid cancer [272]. Similar conclusions were drawn in another study in people with thyroid cancer, where a greater stage of cancer was likewise linked to a higher Cd level [273]. It was also proven that Cd in PTC cells induces heme oxygenase-1 (*HO-1*) and leads to an increase in the level of p21, a cyclin-dependent kinase inhibitor. Increased *HO-1* and p21 expression causes PTC cells to become resistant to apoptotic stimuli and to stop developing during the G0/G1 phase of the cell cycle [274]. However, Cd may cause cancer cells to undergo apoptosis in anaplastic thyroid cancer. There is a positive association between the rise in Cd content and its cytotoxicity. Increased intracellular Ca^2+^ levels linked to Cd-induced cell death activate the *PI3K/AKT*, *p53*, *MAPK*, *G1blok* signaling pathway, mitochondrial dysfunction, caspase-3 activation, and *PARP* cleavage [275].

However, in another study, it was discovered that due to the increase in intracellular Ca^2+^ and the *ERK1/2* activation, Cd in ARO cells induces functional MT1 and MT2 isoforms, which leads to cell cycle transition from the G1 phase to the S phase and the extension of the G2–M phase. This could have an impact on the growth and proliferation of anaplastic thyroid cancer cells [276]. Cd is considered a metalloestrogen, and its action resembles 17β-estradiol. Previous studies have established that estrogen may influence the onset and development of thyroid cancer, so we investigated whether Cd could similarly affect thyroid cancer cells. Estrogen is bound by the G protein-coupled estrogen receptor (*GPER*), which becomes activated. This results in mechanisms that subsequently involve the invasion, metastasis, and proliferation of cell lines of different types of cancer. *GPER* expression was validated in the study on the impact of Cd on human WRO and anaplastic FRO thyroid cancer cells. It has been demonstrated that the effects of Cd on WRO and FRO thyroid cancer cells vary with concentration. At lower concentrations (250 and 500 nM), it enhanced cell survival and number as well as invasion and migration. However, higher Cd concentrations (750 and 1000 nM) led to the inhibition of WRO and FRO cell proliferation, invasion, and migration. It has been proven that the *GPER/ERK&AKT/NF-κB* signaling pathway is engaged in this process. Cd in GPER-positive thyroid cancer cells leads to activation of the *ERK/AKT* pathway, which causes *NF-κB* nuclear translocation, then increases the expression of cyclin A and D1, which are involved in cell proliferation, and IL-8 secretion, which plays a role in migration and invasion [277].

### 3.9. Mercury

Hg is a heavy metal, which may occur in the environment in multiple chemical forms, including metallic elemental Hg (Hg0), inorganic Hg (Hg^2+^), MeHg, and different organic molecules [278]. Both human and natural activities have an impact on the amounts of Hg in the atmosphere and water bodies. Volcanic eruptions are among the natural sources of Hg, whereas industrial operations, small-scale and artisanal gold mining, and the burning of fossil fuels are examples of anthropogenic sources [279,280]. Thyroid disorders have been linked to consuming shellfish and marine seafood. Recent findings indicate that rice consumers are exposed to MeHg in addition to marine foods [279]. Among marine fish species, tuna had the highest Hg content. Samples of hake, pollock, and salmon had the lowest Hg levels [281]. According to the British Food Standards Agency (FSA), children under the age of 16, pregnant women, and women of childbearing age should refrain from eating swordfish and tuna because these higher-ranking ocean fish have higher Hg levels. Additionally, pregnant women and women of childbearing age should refrain from eating more than two tuna steaks per week [282]. If consumed orally, inorganic Hg has a limited bioavailability; depending on the quantity ingested, absorption rates might range from 7% to 15%. MeHg accumulates over time, demethylates to inorganic Hg, and becomes lodged in various organs such as the thyroid, muscles, and breast. MeHg has a half-life of about 70 days in humans. About 90% of it is eliminated through the feces, but some is also carried by the enterohepatic circulation [278].

#### 3.9.1. Mercury and Hashimoto’s Thyroiditis

In a mechanism of molecular mimicry, in which immune responses target the body’s antigens because they resemble structures containing Hg, exposure to Hg can result in the development of autoimmune disorders. A 2014 study sought to determine whether dental amalgam implantation and the autoimmune condition Hashimoto’s thyroiditis are related. It is acknowledged that dental amalgam fillings, which contain roughly 50% Hg, can expose people to Hg [283]. The study was conducted on a study group of 363 patients with Hashimoto’s thyroiditis and a control group of 365 healthy patients. Dental amalgam fillings were checked, and thyroiditis was diagnosed using various criteria. The incidence of dental amalgam implantation did not significantly differ between the group with Hashimoto’s thyroiditis and controls, according to the results. While some studies suggest that Hg from amalgam may play a role in autoimmune thyroiditis, this study did not find a direct relationship. The study concluded that dental amalgam is not a causative factor for Hashimoto’s thyroiditis. Limitations of the study include not evaluating fecal and urine levels of Hg to represent a metal release from amalgam implants. Overall, the study did not support a link between dental amalgam and autoimmune thyroiditis [284].

#### 3.9.2. Mercury and Hypothyroidism

Correia et al. [285] evaluated the effects of long-term occupational metallic Hg exposure on hormonal function, including hypothyroidism. The metal’s deposition in the gland may be facilitated by the extended exposure to Hg and its strong affinity for selenium. According to the study, the mean TSH levels were higher in the group exposed to Hg. Moreover, this difference was statistically significant. The authors suspect that this is due to the inhibition of deiodinase by Hg. These enzymes are essential for the anterior pituitary and other organs’ conversion of T4 to T3. A reduction in T3 can stimulate the pituitary gland, leading to increased TSH secretion. Since there was no statistically significant difference in the percentage of positive antibodies between the groups, autoimmune thyroiditis was not accepted as the reason for the rise in TSH [285].

#### 3.9.3. Mercury and Autoimmune Thyroiditis

Hg may also influence the occurrence of autoimmune thyroiditis. A 2011 study explored the correlation between exposure to Hg and the presence of thyroid autoantibodies in US women, specifically focusing on TgAb and TPOAb. According to the study, Tg antibodies were 2.24 times more common in women with higher blood Hg levels (>1.81 μg/L) than in those with the lowest values (≤0.40 μg/L). This relationship was statistically significant (*p* = 0.032). This relationship was not observed for TPOAb: no significant association was found between blood Hg levels and the presence of TPOAb. The study suggests that Hg exposure may be associated with autoimmune responses directed against Tg, which may have implications for understanding the effects of Hg on autoimmune diseases [286].

#### 3.9.4. Mercury and Thyroid Nodules

The thyroid parenchyma’s structure can change significantly as a result of long-term exposure to Hg. The study by Correia et al. [285] confirmed modifications in the thyroid gland echogenicity. In the Hg-exposed group, a higher incidence of thyroid echogenicity changes was found. Thyroid nodules were similar in both groups; however, features suspected of malignancy were found in 7.2% of those in the Hg-exposed group and 3.6% in the un-exposed group. PTCs were documented in three individuals who were exposed. However, it was not possible to establish an association between the presence of malignant nodules and Hg exposure in the present study [285].

#### 3.9.5. Mercury and Thyroid Cancer

Thyroid cancer may develop as a result of exposure to MeHg. The impact of MeHg on non-tumorigenic thyroid cells was investigated in a 2020 study. It was found that low doses of MeHg can promote cell proliferation through the *ERK* pathway activation. Exposure to MeHg at higher concentrations leads to a decrease in cell viability, while lower concentrations increase cell viability and the G2/M phase. Additionally, the study demonstrated that extended exposure to low concentrations of MeHg promoted cell proliferation. This implies that environmental pollutants such as Hg may influence cell proliferation via the *ERK*-mediated pathway, hence contributing to the prevalence of thyroid cancer. Hg can affect a number of molecular pathways associated with carcinogenesis, including *RAS*, *MAPK*, and *PI3K* signaling, and can cause the formation of ROS, which can damage DNA, inhibit apoptosis, and stimulate cell proliferation. What is more, Hg disrupts the HPT axis, leading to elevated TSH levels and promoting thyroid cell proliferation, and unfortunately, there is a risk that the cells will undergo cancer transformation. The molecular processes behind MeHg’s impact on thyroid cells and carcinogenesis require further study [280].

#### 3.9.6. Mercury and Postpartum Thyroiditis

Consuming tainted seafood has been linked in certain studies to thyroid problems. Benvenga et al. [287] examined the impact of different fish types on thyroid autoimmunity during pregnancy and postpartum. Positive thyroid autoantibody in the serum is a predictor of postpartum thyroiditis. In contrast to swordfish, which can concentrate contaminants like mercury, it is hypothesized that a consistent intake of omega-3-rich oily fish is linked to a more favorable profile of serum thyroid antibodies [287]. A positive association was discovered between antibody levels and swordfish consumption, while an inverse correlation was observed with oily fish consumption. Similar conclusions were found in a 2018 research; women who consumed stable swordfish had higher levels of thyroid antibodies (TPOAb and TgAb) and were more likely to develop postpartum thyroiditis [288]. Overall, these findings suggest dietary recommendations for pregnant women to avoid swordfish and prefer oily fish to prevent postpartum thyroiditis. However, the profile of serum indicators of thyroid autoimmunity is better in women who used to eat swordfish than in women who do not eat fish (meat eaters) [287,288].

### 3.10. Lead

Pb exposure in humans may occur by direct skin contact, consumption of tainted food or water, or breathing of Pb dust-contaminated air [289,290]. Eating habits, especially seafood consumption, were significantly correlated with Pb accumulation in the human body. Pb that reaches the bloodstream has a median biological half-life of about 30 days and is removed through bile and urine. With a half-life of roughly 25 to 30 years, the remaining Pb diffuses into the body’s soft tissues before building up in the bones [289,291]. Pb, like Hg, affects almost all systems: cardiovascular, endocrine, nervous, and many others [292,293,294].

#### 3.10.1. Lead and Hashimoto’s Thyroiditis

A 2020 study showed for the first time a close link between toxic and essential trace elements and Hashimoto’s disease by analyzing samples of thyroid, blood, and urine tissue. Differences in elemental profiles were found between samples from Hashimoto’s disease patients and controls. Key findings included elevated levels of Pb and also As in blood samples and thyroid tissue from Hashimoto’s disease patients. Negative correlations were found between Pb and As with Se, suggesting that Pb and arsenic may inhibit selenium’s presence in thyroid tissue. This research offers novel perspectives on the potential impact of environmental contaminants on thyroid health, specifically in the context of autoimmune thyroid diseases like Hashimoto’s thyroiditis [295].

#### 3.10.2. Lead and Hypothyroidism

Pb concentrations in women were associated with higher TSH levels and hypothyroid status. The study suggests that Pb may affect thyroid function through autoimmune mechanisms, which in turn may lead to hypothyroidism [262]. Low levels of TH are a hallmark of hypothyroidism, a serious endocrine disorder. A meta-analysis of 32 observational studies by Talebi et al. [65] emphasized the importance of microelements in the functioning of the thyroid gland and suggests that Pb excess may influence the development of hypothyroidism [65]. The effect of Pb on thyroid functions in hypothyroid patients was similarly supported by another study. The study’s main objective was to assess how Ca and Pb interact and affect thyroid function in people with hyperthyroidism and hypothyroidism. Serum samples from both kinds of thyroid patients had greater Pb levels [296]. The difference was especially significant in hypothyroid patients compared to the controls. The mean levels of Pb and TSH are significantly higher in patients with hypothyroidism. Further research is recommended to confirm these results and to better understand the mechanisms that may underlie these relationships [56,88,297].

#### 3.10.3. Lead and Autoimmune Thyroiditis

The effect of Pb on autoimmune thyroiditis is not clear. Nie et al. [262] investigated how exposure to Pb affected thyroid function and thyroid antibody levels in a Chinese women population. The purpose of the study was to ascertain whether blood Pb concentrations are associated with antithyroid antibody levels and thyroid dysfunction in the general population. The natural logarithms of Pb (lnBPb) concentrations were found to be positively associated with TPOAb and TSH levels in women. This suggests that Pb may induce thyroid autoimmunity [262]. The opposite conclusion was drawn by Rivera-Buse et al. [298]: they discovered no correlation between elevated blood Pb levels and thyroid antibody, FT4, or TSH levels [298].

#### 3.10.4. Lead and Thyroid Nodules

The connection between thyroid nodules and blood Pb levels has been the subject of a few investigations. Elevated blood Pb levels were correlated with a greater risk for thyroid nodules in the overall population. Larger thyroid glands and more significant thyroid nodules were observed in individuals from high Pb exposure areas compared to those from low Pb exposure areas. This suggests an anatomical impact of Pb exposure on the thyroid. Although underlying processes are still unclear, thyroid nodule growth and progression may be facilitated by Pb-induced immune system disruption and changed thyroid hormone levels [298,299].

#### 3.10.5. Lead and Thyroid Cancer

Based on research to date, it has been proposed that increased Pb concentrations are linked to an increased risk of thyroid disease, including thyroid cancer [88,234,300]. The study in China found that elevated levels of Pb in urine are significantly linked with an increased PTC risk. The precise biological mechanisms linking Pb exposure to the risk of PTC remain uncertain. The explanation is likely a disruption of the function of the HPT caused by Pb [301]. Some studies have reported no marked relationship between Pb levels and thyroid cancer stage or multifocality [272]. Furthermore, the PTC group showed a negative association between Pb levels and TSH and a positive link between T3 and Pb levels at high doses [300].

### 3.11. Selenium

Se is an element that has several functions in the human body but is not physiologically active by itself. DNA synthesis, a basic biological process, requires it as a component of selenocysteine and selenomethionine. Every soil layer contains Se, and while the amount of Se in different items might vary, the primary sources include grain products, meat, offal (liver and kidney), eggs, and fish [302].

#### 3.11.1. Selenium and Graves’ Disease

Se deficiency is a risk factor for Graves’ disease. Se deficiency contributes to the pathophysiology of Graves’ disease by aggravating oxidative stress in thyrotoxicosis and compromising the antioxidant system’s ability to respond to ROS [303]. The 2022 study suggests that serum Se levels were significantly reduced in people with Graves’ disease compared to a healthy control group [304]. Because of this, Se supplementation appears to be useful in the treatment of Graves’ disease. However, as suggested by the 2019 meta-analysis, an in-depth analysis of relevant long-term clinical measures of treatment success is lacking [305]. According to a different meta-analysis, Se treatment significantly reduced FT4 and FT3 levels. Compared to the control group, the selenium-taking group had higher TSH levels. TRAb levels dropped as well. Thyroid function was more likely to improve in individuals receiving Se supplements than in controls. Interestingly, all the above-described effects were detected by the ninth month of Se supplementation [306]. Moreover, additional benefits of Se supplementation appear to occur when treated with methimazole. A study of 103 children who were divided into a control group treated only with methimazole and a study group treated with methimazole and Se showed that treatment was more effective in the bandana group: thyroid volume was significantly lower; IL-6, IL-8, TRAb, TPOAb, and FT4 levels were significantly lower; and it took less time to return to normal levels compared to the control group [307]. Gallo D et al. [308] found similar outcomes when using a combination of methimazole and Se. Interestingly, in vitro experiments in this study demonstrated a substantial decrease in mRNA levels and protein expression of TRAb, TPOAb, and TGA [308]. The use of Se in the alleviation of Graves’ disease itself appears to be a promising line of research. On the other hand, a beneficial effect of Se supplementation on inhibiting the development of orbitopathy in the course of Graves’ disease has been reported. As in the case of Graves’ disease, in orbitopathy, Se deficiency affects the oxidative balance in orbital fibroblasts, causing their proliferation. Due to this, Se supplementation has been shown to reduce the severity of symptoms in orbitopathy and slow the progression to more severe forms of the disease [309,310].

#### 3.11.2. Selenium and Hashimoto’s Thyroiditis

Se deficiency also affects Hashimoto’s thyroiditis. A 2022 study suggested that Se levels were lower in people with Hashimoto’s thyroiditis compared to a healthy control group [307]. Moreover, Se deficiency in Hashimoto’s thyroiditis may exacerbate autoimmune reactions and accelerate the deterioration of thyroid function through oxidative stress [311]. For this reason, Se supplementation therefore seems to be a useful treatment for Hashimoto’s thyroiditis. This conclusion was reached in the 2024 meta-analysis, showing a decrease in levels of TSH, TPOAb, and oxidant markers such as malondialdehyde after Se supplementation in the group with thyroid hormone supplementation compared to the control group of Hashimoto’s thyroiditis patients without thyroid hormone supplementation [312]. Additionally, 90 individuals with Hashimoto’s thyroiditis participated in a study that demonstrated the positive effects of Se supplementation. Se supplementation significantly reduced the levels of TPOAb, TGAb, and TSH in a study group of 47 participants. Furthermore, increased production of GPX3 and selenoprotein P1 in thyrocytes resulted in an increase in antioxidant activity, which could improve thyroid cell function. Kong XQ et al. [313] conducted a meta-analysis and found similar results, showing that Se supplementation reduces blood TPOAb and TgAb levels in patients with Hashimoto’s thyroiditis after 6 months of therapy [313].

#### 3.11.3. Selenium and Autoimmune Thyroiditis

Se supplementation prevents autoimmune thyroiditis, because it may be associated with Se deficiency. A systematic review of 2023 shows that in the autoimmune thyroiditis patient population treated with levothyroxine and Se, TPOAb levels decreased, similar to the group not treated with levothyroxine. In addition, there was also a decrease in TgAb in this group [314]. A study in a rat model also demonstrated the protective role of Se supplementation in the development of autoimmune thyroiditis [315].

#### 3.11.4. Selenium and Thyroid Cancer

The occurrence of nodular goiter may be associated with low Se levels. According to the study, serum Se levels were reduced in the study group with nodular goiter compared to the healthy study group. For this reason, an adequate supply of Se may prevent the formation of thyroid nodules [94]. Se concentrations also appeared to be reduced in patients with thyroid cancer. The 2024 study showed that Se levels were lower among patients with PTC compared to patients with benign thyroid nodules and healthy controls. It is interesting to note that the stage of thyroid cancer was negatively related to blood Se levels [316]. Low serum Se levels encouraged the development of tumor cells, whereas high serum Se levels were cytotoxic and prevented the formation of tumor cells. TSH levels may rise as a result of reduced thyroid hormone production brought on by a Se deficiency. TSH may stimulate the growth of thyroid cells, and chronically elevated TSH levels may cause and encourage the development of thyroid cancer. Consequently, low Se levels may indirectly encourage the rise in TSH and contribute to the formation and incidence of tumors. In contrast, high serum Se concentrations may be a protective factor in patients with PTC. With high selenium concentrations, they were negatively correlated with bilateral and multifocal tumors. A potential mechanism is the anti-tumor effect of Se through inhibition of the ROS-dependent Akt/mTOR pathway, induction of apoptosis through generation of superoxides in mitochondria and activation of the mitochondrial apoptosis pathway, and Se inhibition of tumor cell proliferation through DNA repair by p53-dependent effectors [317]. Se in the form of nanoparticles can also be used as a drug delivery system. An in vivo study showed that they had the ability to efficiently accumulate in the tumor area, thereby increasing the ability to kill tumor cells, as well as low toxicity to other organs. Because of these properties, they appear to be useful in the treatment of thyroid cancer [318]. Se’s radioprotective impact on salivary glands during I-131 treatment is another possible application of Se in thyroid cancer treatment. Se supplementation during I-131 therapy was successful in minimizing radiation-induced damage to the salivary glands, according to a 2017 study that included 16 patients [319].

#### 3.11.5. Selenium and Postpartum Thyroiditis

In the case of postpartum thyroiditis, Se supplementation is also beneficial. A study of 2143 pregnant women found that selenomethionine supplementation at a dose of 200 micrograms per day resulted in a significantly lower incidence of postpartum thyroiditis compared to a control group without supplementation. Additionally, this supplementation reduced TPOAb levels, enhanced the thyroid gland’s echogenic pattern, and had an anti-inflammatory effect [320]. The review from 2022 showed similar results, stating that by lowering levels of TPOAb and TgAb, Se supplementation decreased the risk of thyroid malfunction and persistent hypothyroidism in the postnatal period [321].

## 4. Conclusions

Thyroid gland homeostasis and disease are significantly influenced by trace elements, which are not well understood. Many elements, like Fe, I, Cu, Zn, and Se, are important for normal thyroid functioning. Co, Fe, I, Mn, Zn, Cd, Pb, Hg, and Se are connected with the development of hypothyroidism. I and Cd precipitate in Graves’ disease. Heavy metals (Pb, Hg, and Cd) can damage thyroid cells, impair hormone production, and promote carcinogenesis. Co and Mn compounds have been shown to negatively impact thyroid cancer cells. Further research into the role of trace elements is required. The influence of individual elements on thyroid diseases is summarized in Table 2 and Table 3.

## Figures and Tables

**Table 1:** The influence of trace elements on TH synthesis and metabolism.

Trace Element	Mechanism	Influence on TH Synthesis/Metabolism	Additional Information
Iron (Fe) [26,31]	Builds the thyroperoxidase (TPO).	Fe levels were positively correlated with free forms of triiodothyronine (FT3) and free forms of thyroxine (FT4) and negatively with thyrotropin (TSH).	TPO catalyzes the H_2_O_2_-dependent oxidation reactions of iodide to the iodonium intermediate compound.
Copper (Cu) [31]	Control calcium levels in the body.	Stimulation of thyroxine (T4) production and inhibition of excessive T4 absorption in blood cells.	Data on the exact effects on blood TSH, triiodothyronine (T3), and T4 levels are not consistent.
Cobalt (Co) [35]	Impairs the uptake of I by the thyroid gland. Inhibition of the extrathyroidal 5′-deiodination of T4 into T3.	Increase in FT4 and a decrease in FT3.	-
Iodine (I) [38,39,40]	I is part of the TH.	Increase in TH synthesis.	-
	Increased expression of the X-box binding protein 1 (*XBP1*) gene. *XBP1* inhibits the transcription of genes involved in TH synthesis.	Excess I inhibits TH synthesis.	The mechanism described may be responsible for the Wolff–Chaikoff effect.
Manganese (Mn) [39]	Regulation of hepatic deiodinase activity.	Mn deficiency results in an increase in conversion of T4 to T3.	-
Zinc (Zn) [40]	Regulation of hepatic deiodinase activity.	Zn deficiency results in a decrease in conversion of T4 to T3.	-
Silver (Ag) [43]	Silver nanoparticles (AgNPs) increased the expression of iodothyronine deiodinase 3 (DIO3) mRNA in the thyroid gland.	TH degradation.	-
	AgNPs upregulate DIO2 expression in the liver.	Increase in conversion of T4 to T3.	-
Cadmium (Cd) [44]	Regulation of hepatic 5′-monodeiodinase (5′-D) activity.	Cd inhibits the conversion of T4 to T3.	-
	Cd exposure induces UGT activity in the liver.	Increased T3 and T4 metabolism and a decrease in their plasma concentrations.	-
Mercury (Hg) [9,47,48,49]	Hg in the form of methylmercury (MeHg) interferes with TSH production. Inorganic Hg compounds inhibit TPO production. MeHg and inorganic compounds inhibit Tg iodination.	Decrease in TH concentrations.	-
Lead (Pb) [48]	Regulation of deiodination of T4 to T3.	Cd inhibits the conversion of T4 to T3.	-
Selenium (Se) [24]	Builds deiodinases (DIOs): DIO1, DIO2, and DIO3.	DIO1 is involved in the conversion of T4 to T3 and rT3 and T3 to rT3 or T2; DIO2 is involved in the conversion of T4 to T3 and T3 to T2, and DIO3 is involved in the conversion of T4 and T3 to rT3 and T2	-

FT3—free forms of triiodothyronine; FT4—free forms of thyroxine; TPO—thyroperoxidase; TSH—thyrotropin; TH—thyroid hormone; T4—thyroxine; T3—triiodothyronine; XBP1—X-box binding protein 1; AgNPs—silver nanoparticles; DIOs—deiodinases; 5′-D—5′-monodeiodinase; Fe—iron; Cu—copper; Co—cobalt; I—iodine; Mn—manganese; Zn—zinc; Ag—silver; Cd—cadmium; Hg—mercury; MeHg—methylmercury; Pb—lead; Se—selenium.

**Table 2:** Potential role of trace elements in thyroid disease development.

Trace Element	Thyroid Disease	Mechanism of Influence	Additional Information
Iron (Fe) [27,28,64,68,69,71,72]	Graves’ disease	Patients with ophthalmopathy have shown increased Fe deposition in the brain, which may be responsible for visual, emotional, and cognitive deficits.	-
	Hypothyroidism	Adequate Fe levels are required for the effectively function of thyroid peroxidase (TPO).	-
	Autoimmune thyroiditis (AIT)	Fe deficiency may reduce the activity of TPO and 5′-deiodinase and inhibit T3 with its nuclear receptor, thereby affecting the slower release of T3 from serum. Deficiency increases the risk of autoimmune diseases.	-
	Thyroid nodules	The role of Fe in thyroid nodules is unclear.	-
	Thyroid cancer	-	Hepcidin secreted by thyroid cancer cells, thereby increasing intracellular Fe concentrations, may facilitate tumor cell proliferation.
Copper (Cu) [80,81,83]	Graves’ disease	-	Increased serum Cu levels appear to be positively associated with the occurrence of hyperthyroidism.
	Hashimoto’s thyroiditis	Dysfunction of the Cu/Zn SOD enzyme may be responsible for the oxoreductive imbalance, which may lead to Hashimoto’s thyroiditis, but this concept is uncertain.	-
	Thyroid cancer	It is thought that Cu may initiate angiogenesis in tumor cells.	-
Cobalt (Co) [115,116,124]	Hypothyroidism	The thyroid gland’s ability to absorb I is reversibly reduced by Co. Patients with head and neck cancers treated with external beam radiation using 60 Co have been reported to develop hypothyroidism.	The risk of developing a Co contact allergy is increased by hypothyroidism.
Iodine (I) [133,135,144,145,146,147,148,150,173,174,175,176,178,179,180,186,194,198,208,209,212,213,214,215,216]	Graves’ disease	Increased dietary I levels are associated with an increase in the incidence of Graves’ disease among young people.	-
	Hyperthyroidism	Excess I causes the development of the I-based phenomenon.	-
	Hypothyroidism	Excess I causes the development of the phenomenon Wolff–Chaikoff effect (WCE).	-
	Hashimoto’s thyroiditis	Excess I: (1) Increased pyroptosis activity in thyroid follicular cells by reactive oxygen species-nuclear factor kappa B (*ROS-NF-κB*) signaling pathway and activation of the nucleotide-binding domain, leucine-rich–containing family, pyrin domain–containing-3 (*NLRP3*) and release interleukin 1β (IL-1β). (2) Promotes follicular thyroid cancer (FTC) apoptosis by activating the hypoxia-inducible factor 1 alpha (HIF-1α)-mediated hypoxia signalling pathway and increasing *N*-myc downstream regulated 1 (*NDRG1*) expression and inhibits FTC proliferation in a dose-dependent manner. (3) Can cause increased ROS synthesis and suppression of autophagy activity in thyroid follicular cells through downregulation of transforming growth factor β (TGF-β) and activation of the protein kinase B/mammalian target of rapamycin (*AKT/mTOR*) signaling pathway.	In mice, I promotes the polarization of naive T cells in the spleen into Th17 cells, and extremely high levels of I enhance Th1 polarization and inhibit regulatory t cells (Treg) development. I supplementation during pregnancy does not increase TPO antibodies and does not adversely affect the course of Hashimoto’s disease.
	AIT	I increases the risk of AIT through higher methylation of tyrosine 3 monooxygenase/tryptophan 5-monooxygenase (*YWHAG*) and brain-selective kinase 2 (*BRSK2*) with lower methylation of inhibitor of growth 4 (*ING4*). Excess I causes hypomethylation of most of the CpG locus Ras-related protein Rab-8A (*RAB8A*), olfactory receptor family 4 subfamily K member 17 (*OR4K17*), Ras-related protein Rap-1A (*RAP1A*). I can affect the DNA methylation levels of protein kinase AMP-activated catalytic subunit alpha 2 (*PRKAA2*) and integrin subunit alpha 6 (*ITGA6*) genes. In AIT was observed a concentration-dependent effect of I on death-associated protein kinase 1 (*DAPK1*) and tumor necrosis factor alpha-induced protein 8 (*TNFAIP8*) gene methylation.	Studies in mice showed that (1) excess I causes infiltration of the thyroid gland by Th9 cells, increased expression of IL-9 mRNA and protein, and key Th9 cell transcription factors; (2) I enhanced the spleen’s Th1 and Th17 cell count and accumulation; (3) excess I causes an increase in the number of Th17 cells and IL-17, IL-23, IL-6, and TGF-β expression; (4) the expression of retinoic acid-related orphan receptor gamma t (RORγt), retinoid-related orphan receptor alpha (RORα), signal transducer and activator of transcription 3 (STAT3) was significantly higher, but the expression of forkhead/winged helix transcription factor p3 (Foxp3) was significantly lower.
	Thyroid nodules	When there is a lack of I, the thyroid gland is continuously stimulated, thyroid hormone production decreases, and thyroid-stimulating hormone (TSH) secretion increases.	-
	Thyroid cancer	There are conflicting studies regarding the effect of I levels on the incidence of B-Raf proto-oncogene, serine/threonine kinase (BRAF) mutations. Some studies indicate that there are no differences in *BRAF* mutations depending on I levels, while others show that low or excessive I intake is a risk factor for *BRAF* mutations in papillary thyroid cancer (PTC) in I-rich areas. I deficiency is linked with an increase in the incidence of rat sarcoma virus (*RAS*) mutations, which affect the initiation and/or maintenance of follicular cancers, while another study found no differences in the incidence of rearranged during transfection (RET)/PTC or *RAS* mutations between I-rich and I-poor areas. I at low concentrations activated the *AKT* and extracellular signal-regulated kinase (*ERK*) signaling pathway and caused an increase in the number of migrating thyroid cancer cells. However, I at high concentrations inhibited the *AKT* and *ERK* signaling pathway and reduced the number of migrating thyroid cancer cells. Excess I increased the activation of *AKT* phosphorylation and the expression of phospho-Wee1 (Ser642), while decreasing the expression of phospho-cyclin dependent kinase 1 (CDK1) (Tyr15), inducing the proliferation of thyroid cancer cells. Exposure of cells to I is associated with higher expression of sperm protein associated with the nucleus, X-linked (*SPANXA1*), and increased expression of phosphoinositide 3-kinase (*PI3K*) and *p*-*AKT*.	A study in rats found a protective effect of I by inhibiting BRAF V600E-induced activation of the neurogenic locus notch homolog protein/microRNA 19a (Notch/miR-19) loop and restoring sensitivity to TGF-β signaling.
Manganese (Mn) [2,233,235]	Hypothyroidism	High concentrations of Mn can reduce levels of free T3 and free T4, leading to hypothyroidism. It can also be caused by oxidative stress in the regulation of which Mn is involved.	-
	Thyroid cancer	Mn can be used as an indicator of cancer development and progression, as it shows a significant association with thyroid tumorigenesis in single-element models.	-
Zinc (Zn) [49,94]	Hypothyroidism	Insufficient Zn supplementation causes hypothyroidism.	-
	Thyroid nodules	A Zn deficit may be linked to the development of thyroid nodules as well as nodular goiter of the thyroid gland.	-
Cadmium (Cd) [263,264,265,266,274,276,277]	Hypothyroidism	Cd reduces the surface area of follicles and increases the height of the follicular epithelium. It also causes apoptosis of follicle epithelial cells by activating the extracellular-signal-regulated kinase (*ERK1/2*) pathway, inducing endoplasmic reticulum stress, and by inducing ROS production and lipid peroxidation. Cd can also lead to pyroptosis of thyroid cells by regulating the nuclear factor erythroid 2–related factor 2/Kelch-like ECH-associated protein 1/nuclear factor kappa B (*Nrf2/Keap1/NF-κB*) pathway.	-
	Hashimoto’s thyroiditis	Monocyte chemoattractant protein-1 and the CXC motif chemokine 10 are both expressed in response to Cd. Inflammatory cytokines like IL-1β, IL-6, and tumor necrosis factor-alpha (TNF-α) were produced in greater quantities when Cd was present.	-
	PTC	Cd increases heme oxygenase-1 and p21 expression.	-
	Anaplastic thyroid cancer	Cd induces cancer cell proliferation and growth by activating *ERK1/2* and via the G protein-coupled estrogen receptor (*GPER*)/*ERK*&*AKT*/*NF-κB* signaling pathway.	It also has a positive impact, which is described in Table 3.
	FTC	Cd induces tumor cell proliferation, invasion, and migration via the GPER/ERK&AKT/NF-κB signaling pathway.	-
Mercury (Hg) [280,285,286,287]	Hypothyroidism	High concentrations of Hg can increase the level of TSH, leading to hypothyroidism. It is probably caused by inhibition of deiodinase.	-
	AIT	Women with higher blood Hg levels were 2.24 times more likely to have thyroglobulin antibodies compared to women with the lowest levels of Hg.	-
	Thyroid nodules	Hg accumulates in the thyroid gland.	In the Hg-exposed group, a higher incidence of thyroid echogenicity changes was found.
	Thyroid cancer	By activating the *ERK* pathway and *RAS*, *MAPK*, and *PI3K* signaling, methylmercury (MeHg) can stimulate cell proliferation. It can also generate reactive oxygen species (ROS), which can damage DNA, prevent apoptosis, and promote cell proliferation. Hg disrupts the HPT axis, leading to elevated TSH levels and promoting thyroid cell proliferation.	-
	Postpartum thyroiditis	A positive association was discovered between antibody levels and swordfish (which can concentrate pollutants like Hg) consumption.	-
Lead (Pb) [56,88,262,272,297,298,299,301]	Hashimoto’s thyroiditis	Negative correlations were found between Pb with Se, suggesting that Pb may inhibit selenium’s presence in thyroid tissue.	-
	Hypothyroidism	Pb concentrations in women were associated with higher TSH levels and hypothyroid status.	-
	AIT	Pb concentrations are associated with antithyroid antibody levels and thyroid dysfunction.	-
	Thyroid nodules	A higher risk of thyroid nodules was linked to elevated blood Pb levels, although the underlying mechanisms are still unclear.	According to certain research, there is no discernible correlation between Pb levels and the stage or multifactorial nature of thyroid cancer.
	Thyroid cancer	An elevated risk of PTC is substantially correlated with elevated Pb concentrations in urine. The explanation is likely a disruption of the function of the HPT caused by Pb.	-
Selenium (Se) [94,303,304,309,310,311,312,314,315,316,317,320]	Graves’ disease	Se deficiency contributes to the pathophysiology of Graves’ disease by aggravating oxidative stress in thyrotoxicosis and compromising the antioxidant system’s ability to respond to ROS. In orbitopathy, Se deficiency affects the oxidative balance in orbital fibroblasts, causing their proliferation.	-
	Hashimoto’s thyroiditis	Se deficiency in Hashimoto’s thyroiditis may exacerbate autoimmune reactions and accelerate the deterioration of thyroid function through oxidative stress.	-
	AIT	AIT may be associated with Se deficiency.	-
	Thyroid nodules	The occurrence of nodular goiter may be associated with low Se levels.	-
	Thyroid cancer	Low serum Se levels encouraged the development of tumor cells. TSH levels may rise as a result of reduced thyroid hormone production brought on by an Se deficiency. TSH may stimulate the growth of thyroid cells, and chronically elevated TSH levels may cause and encourage the development of thyroid cancer. Consequently, low selenium levels may indirectly encourage the rise in TSH and contribute to the formation and incidence of tumors.	-
	Postpartum thyroiditis	Low Se levels are associated with increased TPOAb and TgAb levels and change in the thyroid gland’s echogenic pattern and have an inflammatory effect.	-

Ag—silver; AIT—autoimmune thyroiditis; AKT—protein kinase B; BRAF—B-Raf proto-oncogene, serine/threonine kinase; BRSK2—brain-selective kinase 2; Cd—cadmium; CDK1—cyclin-dependent kinase 1; Co—cobalt; DAPK1—death-associated protein kinase 1; ERK—extracellular-signal-regulated kinase; Fe—iron; Foxp3—forkhead/winged helix transcription factor p3; FTC—follicular thyroid cancer; GPER—G protein-coupled estrogen receptor; Hg—mercury; HIF-1α—hypoxia-inducible factor 1 alpha; HPT—hypothalamus–pituitary–thyroid axis; hs-CRP—high-sensitivity *C*-reactive protein; I—iodine; IL—interleukin; ING4—inhibitor of growth 4; ITGA6—integrin subunit alpha 6; Keap1—Kelch-like ECH-associated protein 1; MeHg—methylmercury; Mir19A—microRNA 19a; Mn—manganese; mTOR—mammalian target of rapamycin; NDRG1—N-myc downstream regulated 1; NF-κB—nuclear factor kappa-light-chain-enhancer of activated B cells; NLRP3—nucleotide-binding domain, leucine-rich–containing family, pyrin domain–containing-3; Notch—neurogenic locus notch homolog protein; Nrf2—nuclear factor erythroid 2–related factor 2; OR4K17—olfactory receptor family 4 subfamily K member 17; Pb—lead; PI3K—phosphoinositide 3-kinase; PRKAA2—protein kinase AMP-activated catalytic subunit alpha 2; PTC—papillary thyroid cancer; RAB8A—Ras-related protein Rab-8A; RAP1A—Ras-related protein Rap-1A, RAS—rat sarcoma virus; RET—rearranged during transfection; RORα—retinoid-related orphan receptor alpha; RORγt—retinoic acid-related orphan receptor gamma t; ROS—reactive oxygen species; Se—selenium; SPANXA1—sperm protein associated with the nucleus, X-linked; STAT3—signal transducer and activator of transcription 3; TGF-β—transforming growth factor β; TNF-α—tumor necrosis factor-alpha; TNFAIP8—tumor necrosis factor alpha-induced protein 8; Treg—regulatory T cells; TSH—thyroid-stimulating hormone; WCE—Wolff–Chaikoff effect; YWHAG—tyrosine 3-monooxygenase/tryptophan 5-monooxygenase; ZHX2—zinc fingers homeoboxes; Zn—zinc; ZNF703—zinc finger protein 703.

**Table 3:** The positive role of trace elements in thyroid disease.

Trace Element	Thyroid Disease	Mechanism of Influence	Additional Information
Iron (Fe) [73,74,75,77]	Thyroid cancer	-	The phenomenon of ferroptosis is becoming a focus of clinical research for use in anticancer therapies for papillary, follicular, and anaplastic thyroid cancer.
Copper (Cu) [97,98,108]	Thyroid cancer	There is a potential role for cuproptosis and increasing ferredoxin-1 expression in the treatment of thyroid cancer. There are reports that the ratio 65 Cu/63 Cu (δ 65 Cu) may in the future act as a cancer biomarker.	The B-Raf proto-oncogene, serine/threonine kinase (BRAF) mutation involved in Cu-dependent oncogenic signaling is the most common genetic alteration in thyroid cancer.
Cobalt (Co) [70,112,128,129]	Graves’ disease	Co is the element used to treat Graves’ ophthalmopathy in the form of Co irradiation.	-
	Papillary thyroid cancer (PTC)	Co reduces the decreased risk of capsular invasion and lymph node metastasis in papillary thyroid cancer. Co can also be used for external beam radiation treatment in PTC and follicular thyroid cancer (FTC).	-
Iodine (I) [139,206,218]	Graves’ disease	I-131 can be used as a first-line treatment for hyperthyroidism in the course of Graves’ disease.	-
	Thyroid cancer	Low I intake is a protective factor for central lymph nodes (CLNM) in PTC with tumor diameter < 1 cm. I in the form of radioactive I is used in the treatment of thyroid cancer.	-
Manganese (Mn) [237,238]	Thyroid cancer	MnO_2_/CDDP@PDA-Cy5.5 can effectively kill cancer cells in vitro. The creation of HSA-MnO_2_-131I has enabled imaging and anaplastic thyroid cancer treatment.	-
Zinc (Zn) [14,60,245,246]	Hypothyroidism	Zn supplementation significantly reduces serum high-sensitivity *C*-reactive protein (hs-CRP) levels and significantly increases serum free forms of thyroxine (FT4) levels in hypothyroid patients.	-
	Hashimoto’s thyroiditis	Normal thyroid function is restored in patients with Hashimoto’s disease when Zn levels are restored.	-
	Thyroid cancer	Zinc fingers homeoboxes (ZHX2) inhibit thyroid cancer metastasis by inhibiting calcium-binding protein expression at the transcriptional level. Zinc finger protein 703 (ZNF703) may be a key regulator of medullary thyroid cancer development and progression.	-
Silver (Ag) [251,252,253]	PTC	Silver nanoparticles inhibit cell proliferation by increasing p53 levels and induce apoptosis via reactive oxygen species (ROS).	-
Cadmium (Cd) [275]	Anaplastic thyroid cancer	Cd induces apoptosis of cancer cells by activation of phosphoinositide-3 kinase (*PI3K*) and p53.	It also has a negative impact, which is described in Table 1.
Selenium (Se) [94,305,306,307,308,309,310,312,313,314,315,317,318,319,320,321]	Graves’ disease	Se supplementation improves the oxidative balance in thyrocytes and also prevents the formation of orbitopathy in the mechanism of restoring the oxidative balance in orbital fibroblasts.	-
	Hashimoto’s thyroiditis	Se supplementation improves the oxoreductive balance in thyrocytes.	-
	AIT	The protective role of Se supplementation was shown in the development of AIT.	-
	Thyroid cancer	A potential mechanism is the anti-tumor effect of Se through inhibition of the ROS-dependent *AKT/mTOR* pathway, induction of apoptosis through generation of superoxides in mitochondria and activation of the mitochondrial apoptosis pathway, and Se inhibition of tumor cell proliferation through DNA repair by p53-dependent effectors. Se in the form of nanoparticles can also be used as a drug delivery system. Se’s radioprotective impact on salivary glands during I-131 treatment is another possible application of Se in thyroid cancer treatment.	-
	Postpartum thyroiditis	Se supplementation reduced TPOAb levels, enhanced the thyroid gland’s echogenic pattern, and had an anti-inflammatory effect.	-

Ag—silver; BRAF—B-Raf proto-oncogene, serine/threonine kinase; Cd—cadmium; CLNM—central lymph nodes; Co—cobalt; Cu—copper; Fe—iron; FT4—free forms of thyroxine; hs-CRP—high-sensitivity *C*-reactive protein; I—iodine; Mn—manganese; PI3K—phosphoinositide-3 kinase; PTC—papillary thyroid cancer; FTC—follicular thyroid cancer; ROS—reactive oxygen species; Se—selenium; ZHX2—zinc fingers homeoboxes; Zn—zinc; ZNF703—zinc finger protein 703.

## Data Availability

No new data were created or analyzed in this study.

## Abbreviations

AgNPs	Silver nanoparticles;
AIT	Autoimmune thyroiditis;
AKT	Protein kinase B;
ALDH	Aldehyde dehydrogenase;
ALK	Anaplastic lymphoma kinase;
Anti-Tg	Antithyroglobulin;
ART	Artesunate;
ATC	Anaplastic thyroid carcinoma;
ATD	Anti-thyroid drugs;
BMI	Body mass index;
BRAF	B-Raf proto-oncogene, serine/threonine kinase;
BRSK2	Brain-selective Kinase 2;
Cd	Cadmium;
CD4	Cluster of differentiation 4;
CDK1	Cyclin-dependent kinase 1
CLNM	Central lymph nodes;
Co	Cobalt;
CSCs	Cancer stem cells;
CT	Computed tomography;
Cu	Copper;
CXCL10	CXC motif 10 chemokine;
CXCL14	C-X-C motif chemokine ligand 14;
CXCL8	CXC motif chemokine ligand 8;
DAPK1	Death-associated protein kinase 1;
DICER1	Dicer 1 ribonuclease III;
DIOs	Deiodinases;
Dit	Diiodothyronine;
DMPS	2,3-dimercaptopropane-1-sulfonate;
DSF	Disulfiram;
DTC	Differentiated thyroid carcinoma;
DTCs	Differentiated thyroid carcinomas;
DUOX2	Dual oxidase 2;
EBRT	External beam radiotherapy;
EDTA	Ethylenediaminetetraacetic acid;
ERK	Extracellular signal-regulated kinase;
ES	Esclomol;
FDX1	Ferredoxin-1;
Fe	Iron;
Foxp3	Forkhead/winged helix transcription factor p3;
FT3	Free forms of triiodothyronine;
FT4	Free forms of thyroxine;
FTC	Follicular thyroid cancer;
GO	Graves’ ophthalmopathy;
GPER	G protein-coupled estrogen receptor;
GPX4	Glutathione peroxidase 4;
GSH-Px	Glutathione peroxidise;
Hb	Hemoglobin;
HER3	Human epidermal growth factor receptor-3;
Hg	Mercury;
HIF-1α	Hypoxia-inducible factor 1 alpha;
HLA-DPB1	Major histocompatibility complex, class II, DP beta 1;
HO-1	Heme oxygenase-1;
HPT	Hypothalamus–pituitary–thyroid axis;
hs-CRP	High-sensitivity *C*-reactive protein;
I	Iodine;
ICAM1	Intercellular adhesion molecule 1;
ICM	Iodine contrast media;
IFNα	Interferon-α;
IgE	Immunoglobulin E;
IIH	Iodine-induced hyperthyroidism;
IL	Interleukin;
INFγ	Interferon-γ;
ING4	Inhibitor of growth 4;
IRF-4	Interferon regulatory factor 4;
ITGA6	Integrin subunit alpha 6;
JBP	Iodine-based phenomenon;
Keap1	Kelch-like ECH-associated protein 1;
LC3-II	1A/1B-light chain 3—phosphatidylethanolamine conjugate;
LC-AgNPs	Silver nanoparticles from Livistona chinensis leaf extract;
MAPK	Mitogen-activated protein kinase;
MCP-1	Monocyte chemoattractant protein-1;
MCT10	Monocarboxylate transporter 10;
MCT8	Monocarboxylate transporter 8;
MeHg	Methylmercury;
MEK1/2	Mitogen-activated protein kinase;
Mir19A	MicroRNA 19a;
Mit	Monoiodothyronine;
MMPs	Matrix metalloproteins;
Mn	Manganese;
MONPs	Metal oxide nanoparticles;
MRI	Magnetic resonance imaging;
mRNA	Micro-RNA;
MT	Metallothioneins;
MTC	Medullary thyroid carcinoma;
MTH1	Targeting MutT homolog 1;
mTOR	Mammalian target of rapamycin;
MVD	Microvessel density;
NaI	Sodium iodide;
NDRG1	N-myc downstream regulated 1;
NF-κB	Nuclear factor kappa-light-chain-enhancer of activated B cells;
NICU	Neonatal intensive care unit;
NIS	Sodium/iodide symporter;
NKX2 1	NK2 homeobox 1;
NLRP3	Nucleotide-binding domain, leucine-rich–containing family, pyrin domain–containing-3;
Notch	Neurogenic locus notch homolog protein;
Nrf2	Nuclear factor erythroid 2–related factor 2;
NSAIDs	Non-steroidal anti-inflammatory drugs;
OR4K17	Olfactory receptor family 4 subfamily K member 17;
PAX-8	Paired box gene 8;
Pb	Lead;
PDCD1LG2	Programmed cell death 1 ligand 2;
PI3K	Phosphatidylinositol 3-kinase;
PRKAA2	Protein kinase AMP-activated catalytic subunit alpha 2;
Ps-AgNPs	P. aeruginosa synthesized AgNPs;
PTC	Papillary thyroid cancer;
PU.1	Transcription factor PU.1;
RAB8A	Ras-related protein Rab-8A;
RAI	Radioactive iodine;
RAIR	Radioiodine refractory;
RAIR-DTC	Iodine-refractory differentiated thyroid cancer;
RAP1A	Ras-related protein Rap-1A;
RAS	Rat sarcoma virus;
RET	Rearranged during transfection;
RORα	Retinoid-related orphan receptor alpha;
RORγt	Retinoic acid-related orphan receptor gamma t;
ROS	Reactive oxygen species;
rT3	Reverse triiodothyronine;
RTHβ	Resistance to thyroid hormone β;
SCD	Systemic contact dermatitis;
Se	Selenium;
SF	Serum ferritin;
SLC5A5	Solute carrier family 5 member 5;
SOD	Superoxide dismutase;
SPANXA1	Sperm protein associated with the nucleus, X-linked, family member A1;
SPECT	Single photon emission computed tomography;
STAT3	Signal transducer and activator of transcription 3;
sTg	Stimulated thyroglobulin;
T2	3,3′-diiodo-L-thyronine;
T3	Triiodothyronine;
T4	Thyroxine;
TBG	Thyroxine-binding globulin;
TC	Total cholesterol;
TERT	Telomerase reverse transcriptase;
Tg	Thyroglobulin;
TG	Triglyceride;
TgAb	Autoantibodies against thyroglobulin;
TGF-β	transforming growth factor β;
TH	Thyroid hormones;
THA	Total hip arthroplasty;
THCA	Tetrahydrocannabinolic acid;
TIMPs	Tissue inhibitors of matrix metalloproteinases;
TM	Tetrathiomolybdate;
TNF-α	Tumor necrosis factor-alpha;
TNFAIP8	Tumor necrosis factor alpha-induced protein 8;
TP53	Tumor protein p53;
TPO	Thyroperoxidase;
TPOAb	Autoantibodies against thyroid peroxidise;
Treg	Regulatory T cells;
TRH	Thyrotropin-releasing hormone;
TSH	Thyroid-stimulating hormone;
TSHoma	Thyrotropin-secreting adenoma;
TSHR	Thyrotropin receptor;
TTR	Transthyretin;
UIC	Urinary iodine concentration;
UIE	Urinary iodine excretion;
UPS10	Ubiquitin-specific peptidase 10;
USI	Universal salt iodization;
UTC	Undifferentiated thyroid cancer;
VEGF-A	Vascular endothelial growth factor;
WCE	Wolff–Chaikoff effect;
XBP1	X-box binding protein 1;
YWHAG	Tyrosine 3-monooxygenase/tryptophan 5-monooxygenase;
ZHX2	Zinc fingers homeoboxes;
Zn	Zinc;
ZNF703	Zn finger protein;
